# Media amplification under the floodlight: Contextualizing 20 years of US risk news

**DOI:** 10.1111/risa.17701

**Published:** 2025-02-05

**Authors:** Cormac Bryce, Michael Dowling, Suwan (Cheng) Long, Jamie K. Wardman

**Affiliations:** ^1^ Bayes Business School, City St George's University of London London UK; ^2^ DCU Business School Dublin City University Dublin Ireland; ^3^ IESEG School of Management University of Lille, CNRS, UMR 9221 ‐ LEM‐ Lille Economie Management Lille France; ^4^ University of Leicester School of Business University of Leicester Leicester UK

**Keywords:** media, risk communication, social amplification of risk, topic modeling

## Abstract

This paper addresses the question of identifying and distinguishing risk amplification incidents and patterns in the news media. To meet this objective, our study incorporates a novel “floodlight” approach utilizing the Society for Risk Analysis Glossary in conjunction with topic modeling and time‐series analysis, to investigate risk‐focused stories within a corpus of 271,854 US news articles over the past two decades. We find that risk amplification in the US news media is concentrated around seven core risk news categories—business, domestic affairs, entertainment, environment, geopolitics, health, and technology—which also vary in the risk‐related terms that they predominantly employ. We also identify 14 signal events that can be distinguished relative to general risk news within their categories. Across these events, the “War on Terror” and COVID‐19 are seen to display uniquely dynamic media reporting patterns, including a systemic influence between risk news categories and the attenuation of other risk news. We discuss possible explanations for these findings along with their wider research and policy implications.

## 1 INTRODUCTION

News coverage of “once in a generation” events such as the September 11 attacks helps to define our experiential understandings of risk (Slovic, 2020). By raising awareness and capturing society's attention, the mass circulation of risk‐related information and imagery can place an indelible mark on how we learn from major events (Conway et al., 2009; Vyncke et al., 2017), as well as frame how we respond to future threats encountered (Joffe & Bettega, 2003; Joffe & Lee, 2004; Slovic, 1987; Wardman & Mythen, 2016). Studies employing the “Social Amplification of Risk Framework” (SARF) have notably drawn attention to the way the transmission and circulation of risk information is intensified and dampened by the media among other key actors within society (Kasperson et al., 1988; Kasperson & Kasperson, 1996). Researchers have also extended these considerations to include more fine‐grained analyses of the role of key variables such as language, culture, emotion, and politics in the amplification and attenuation of risk before, during, and after major events (Chung & Yun, 2013; Duckett & Busby, 2013; Flynn, 2003; Glik, 2007; Jagiello & Hills, 2018; Masuda & Garvin, 2006; Wardman & Löfstedt, 2018).

Notwithstanding these contributions, some researchers have noted a lack of plain criteria by which to judge if risk amplification has taken place and to what effect (Kasperson & Kasperson, 1996; Pidgeon et al., 2003; Simon & Camargo, 2023; Wardman & Löfstedt, 2018), while others further observe that the broader constitution of risk amplification dynamics and processes remains underexplored (Fellenor et al., 2019; Rayner, 1988; Wardman, 2008). For instance, recent research has highlighted the possibility of a wider spectrum of risk amplification and attenuation patterns, as when extensive broad‐based media coverage acts to amplify risk without an initial single event necessarily serving as the trigger (Duckett et al., 2021), and that risk attenuation processes can be associated with risk amplification (Fjaeran et al., 2024). Such observations have led to calls to incorporate more expansive conceptual understandings and empirical analyses that attend to more variegated forms of risk amplification and attenuation (Fellenor et al., 2019). Yet, to date, little critical attention has generally been afforded to elaborating these features, nor to mapping the unique occurrence of signal events against the wider backdrop of risk news. Subsequently, fundamental questions remain concerning how to meaningfully identify and distinguish different forms and patterns of media risk amplification and attenuation.

We suggest that one potential barrier to addressing these questions may lie in the way that SARF studies have traditionally focused on shining a *spotlight* on specific facets of amplification. Which is to say, SARF research has typically addressed the growth in news attention surrounding “discreet” single‐issue signal events confined to a particular geographical location, or moment in time, without reference to their broader context or relative significance in the wider risk amplification landscape. For instance, a *spotlight* investigative approach is typically narrowly focused on “topic‐specific” search terms (e.g., “zika,” “swine flu,” or “opioid”) when investigating the emergence and amplification of a particular risk topic, event, or incident. This approach can allow for a detailed examination of an issue of concern, but it does not illuminate the relative significance of incidents or events compared to other topics, nor capture the wider dynamics and impacts of risk amplification and attenuation across the news. In some cases, *spotlight* approaches might even be susceptible to inflating the relative importance of single issues and events, by collecting all keyword‐related news articles whether they are risk‐related or not, unless extensive manual filtering is employed to screen out irrelevant news articles.

This paper aims to provide a more refined understanding of the broader contours and dynamics of media risk amplification and attenuation by addressing the wider question of how media attention varies across different risk topics and signal events, respectively, over time. To do so, we introduce a more inclusive *floodlight* approach that incorporates risk‐related terms (e.g., “uncertainty” and “harm”) to identify and compare historic patterns in media reporting of risk across all issues and events over time. Specifically, our keyword search employs terms from the Society for Risk Analysis (SRA) Risk Glossary in tandem with a comprehensive mixed‐methods analysis to examine an English‐language datadset of 271,854 US news articles from 2000 to 2021. By using this broader *floodlight* approach, we are able to discover prominent topics within categories of risk reporting, as well as notable patterns of media amplification and attenuation across all US risk news coverage.

This study makes several key contributions to risk amplification research. First, our *floodlight* approach demonstrates the utility of SRA Risk Glossary keyword terms for collating a large corpus of risk‐related news that encompasses a wide range of risk‐related articles for holistic analysis. Second, we identify that US media risk amplification over the past two decades gravitates around 32 topics from which seven main risk news categories can be derived: business, domestic affairs, entertainment, environment, geopolitics, health, and technology. Third, our findings show that media coverage relating to each category adopts domain‐specific terms, thereby indicating that the lexicon of risk employed in media reporting of risk is both broadly diverse as well as distinguishable and specialized across different risk news contexts. Finally, we also identify 14 risk amplification signal events *within* these categories, including two cases of “cascading media amplification” relating to compounding signal events *between* categories associated with the “War on Terror” and the “COVID‐19 pandemic”. One novel standout feature of interest is the attenuating effect of COVID‐19 on other risk news, which, to the authors' knowledge, is the first time this phenomenon has been empirically identified. We suggest that by helping to substantiate the occurrence of a variety of previously undocumented fundamental media amplification and attenuation phenomena, our study offers an important holistic understanding of media risk amplification that helps to contextualize and extend the findings of prior studies.

The remainder of the paper is structured as follows. In Section 2, we review current research insights regarding risk amplification events and processes in the media, reflecting on the different forms and patterns of media attention exhibited. In Section 3, we introduce our novel mixed‐methods approach and specify the data sampling and analytical strategies which serve as a basis for conducting our *floodlight* comparative risk signal event analysis. Section 4 presents our main study findings. Section 5 discusses these findings along with their implications, our study limitations, and possible future research directions.

## 2 LITERATURE REVIEW

The introduction of SARF in the late 1980s provided an important holistic overview of the key information “processes,” “stations,” and “channels” by which risk events gain public “signal value” and are “amplified,” before generating wider “ripple effects” throughout society (Fife‐Schaw & Rowe, 1996; Frewer et al., 2002; Lachlan et al., 2016; Pidgeon et al., 2003; Reynolds & Seeger, 2005; Jin & Spence, 2021). Specifically, SARF elaborates the interactions that occur between the biological and physical properties of hazards and wider social components, mechanisms, and communication pathways, which are commonly divided into two main stages. In Stage 1, risk amplification is typically understood to begin with a “signal event,” such as the emergence of a novel hazard or threat that characteristically embodies attributes that are intrinsically of concern to observers (Pidgeon et al., 2003). If this initial risk event has strong “signal value,” it can gain social attention and further intensify through interpersonal communication and the exchange of corresponding messages and images that are taken up, filtered, and circulated by “amplification stations,” such as news media organizations (Binder et al., 2014). In Stage 2, “ripple effects” are observed when risk signals resonate and grow, and their associated impacts reverberate throughout society. As the circulation of risk signals becomes more widespread and permeates into different areas of public discourse they can influence the awareness, understanding, and behaviors, including those of the general population alongside government institutions and other organizations (Kasperson et al., 1988; Wardman & Löfstedt, 2018). Ripple effects may also vary in form and magnitude, but in more extreme cases are commonly observed to lead to secondary impacts, such as consumer boycotts and protests, the stigmatization of persons, activities, or products, and the imposition of stricter regulations (Pidgeon et al., 2003). This can result in further risk avoidance and the instigation of protective measures, as well as produce economic losses (Kasperson & Kasperson, 1996). Yet, despite their importance, there is comparatively little research into the connections between risk amplification and ripple effects (Wardman & Löfstedt, 2018), or work exploring the operation of ripple effects across different risk domains and contexts (Cox et al., 2022).

A foremost concern among SARF studies is the pivotal role played by mass media, broadly encompassing newspapers, TV, film, radio, and latterly social media (Binder et al., 2014; Kasperson et al., 1988). As people do not typically encounter exposure to many risks immediately or directly, news outlets have traditionally acted as a primary “amplification station” by way of their central role in gathering, filtering, and circulating risk signals through public channels (Binder et al., 2014; Kasperson et al., 1988; Rossmann et al., 2018; Vasterman, 2005; Vasterman et al., 2005). The importance of the media is underscored by findings showing that a more rapid increase in media messaging is associated with “high dread” risks, which are known to arouse substantial fear and anxiety (Jagiello & Hills, 2018). Research also shows that people's media use is associated with the perceived severity of health hazards (Berger & Milkman, 2012), and that those risk stories which highly resonate with the concerns of news consumers are also more likely to be shared with others, especially if they contain elements which create strong emotional arousal, such as anger, anxiety, and fear (Frewer et al., 2002; Young et al., 2008).

In the recent context of the “COVID‐19 pandemic,” for example, national newspapers quickly mobilized to highlight the risk of what for many outside China started off as a “far‐flung” illness (Fu & Zhu, 2020; Nielsen et al., 2020). As the crisis unfolded, traditional news media became increasingly seen as a vital source of information and opinion about the disease, as people sought to know more about this new threat (Nielsen et al., 2020). Meanwhile, media sources were also found to be a source of misreporting exacerbated in part by factors such as high uncertainty and the pace of changing scientific knowledge, meaning stories often had to be updated, amended, or withdrawn (Cinelli et al., 2020; Krause et al., 2020; Wardman & Lofstedt, 2020; Zarocostas, 2020). Nevertheless, those who obtained their information from traditional news media generally felt better informed about COVID‐19 (Nielsen et al., 2020).

As the “gatekeepers” of news, journalists, editors, and/or media owners determine what to publish taking into consideration how the characteristics of a risk story will shape its “newsworthiness” in light of likely public interest and their own “news agendas” (Vasterman et al., 2005; White, 1950). Newsworthiness is conventionally assumed to correspond to the features of reports and media stories that people find intrinsically interesting and wish to hear about. This can be enhanced by attributes such as the prominence and importance of events, whether there is a human interest angle, if there is any conflict and controversy attached to the issue, and the timeliness and proximity of the events covered (Shoemaker & Reese, 1996). For instance, news media coverage of the A/H1N1 “swine flu” virus outbreak in the Spring 2009 was underpinned by “risk‐amplifying frames” that placed emphasis on conflict and damage, thereby attempting to draw out the “drama” and emotion of the situation to gain public attention (Rossmann et al., 2018).

Studies into natural language uses of the word “risk” also show that it can be variably incorporated into news stories to convey different meanings. One early research study (Hamilton et al., 2007) found that “risk” can be employed both as a noun or a verb, and can incorporate positive or negative semantic prosodies to convey particular ideas, understandings, and perspectives that are reflective of different contexts of everyday life and activity. Interpretations of specific risk terms and concepts may also reflect particular associations and carry different meanings between cultural groups, and their wider significance may, respectively, be independent of what originally occurred or was intended (Kasperson et al., 1988). Consequently, this means that the use and interpretation of risk terms and concepts in news media are perhaps best understood as multidimensional and not simply confined to describing major risk events and their characteristics. For these reasons, the ways in which the media actively selects, interprets, filters, and relays risk information, whether to report things factually or to purposely amplify or dampen concerns, is of special interest (Freudenburg et al., 1996; Jenkins et al., 2018; Pidgeon et al., 2003).

News media reports may, as a consequence, be understood to help cultivate and frame an audience's understandings and appraisals of risk in ways that can serve to raise awareness and understanding as well as give reassurance or conversely cause alarm (Hertwig et al., 2005; Pachur et al., 2012; Trumbo & McComas, 2003). Corporations, NGOs, government agencies, or indeed concerned individuals, thus often seek to increase awareness and gain sympathy by aiming to publicize their version of events with the help of the media (Wardman & Löfstedt, 2018). Consequently, contra to some criticisms, it has been remarked that the amplification of public risk stories is not a passive transfer of publicly derived facts from producers to wider audiences (Wardman & Löfstedt, 2018). Risk amplification studies therefore underscore the central, albeit variegated, contributions of the media to raising public awareness, shaping understandings, offering counterviewpoints, and prompting alarm for emerging and enduring threats (Hertwig et al., 2005; Pachur et al., 2012; Trumbo & McComas, 2003).

### 2.1 Charting media amplification and signal value

SARF has also been applied to explain the volume of news over time, typically with regard to the impacts of differences in news framing and content between media channels and platforms (Freudenburg et al., 1996; Rossmann et al., 2018). The traditional “yardsticks of newsworthiness” (i.e., media interest) broadly equate to the “signal value” of risk stories and by extension the selection, extent, duration, and half‐life of risk news attention and coverage (Binder et al., 2014). In this way, the newsworthiness of risk stories contributes to the overall levels and patterns of amplification reflecting in media coverage, commonly known as “issue attention cycles,” given over to reporting risk (Binder et al., 2014; Wirz et al., 2018). Research to date has generally indicated patterns of risk‐related media coverage broadly exhibit an initial increase over a short period in the beginning, which subsequently decreases over time. Nevertheless, news attention can vary by context and duration, with dramatic “risk events” characteristically the subject of focused coverage that soon drops off, and long‐term or continuous risks being the subject of typically fluctuating news coverage which ebbs and flows (Binder et al., 2014; Wirz et al., 2018).

Risk stories may be particularly enduring and have a longer news cycle if they have a strong narrative, which is to say a “good storyline” that resonates across different social, political, and policy arenas (McInerney et al., 2004), especially if it is high in both newsworthiness and risk signal value (Binder et al., 2014). This has been evidenced in cases such as the controversial sinking of the Brent Spa oil platform (Bakir, 2005). Yet, while the resonance of risk news stories is argued to be key to achieving amplification (Renn, 2011), the relationship between media coverage and levels of public concern is not always considered to be directly proportionate to the severity of the risk in question (Betsch et al., 2020; Chung, 2011; Combs & Slovic, 1979; Lichtenberg & MacLean, 1991), or always to represent a linear causal relationship (Fellenor et al., 2019; Jenkins et al., 2018). For instance, the filtering of risk information by the media may also have an attenuating, or dampening effect, as when news coverage downplays the risk in question or simply does not report on it at all or in any great detail (Kasperson & Kasperson, 1996; Pidgeon et al., 2003). News media outlets can choose not to run reports on a risk story if the “scoop” was obtained by a rival outlet for example.

Prior efforts to chart the spread and impact of information across a variety of media, communication, information, and policy domains have offered some instructive insights in these regards (Freudenburg et al., 1996; Rossmann et al., 2018). For example, health communication studies have commonly employed metrics of *news reach* (size of audience exposure), *share of voice* (share of ink), and *sentiment* (positive, neutral, or negative “tone”) to evaluate health campaigns (Kreps, 2014). New media and “big data” studies have similarly specified the “qualities” of information and data sharing in terms of metrics such as *volume, velocity, and variety* (Wardman, 2017). Public relations research has also developed comparable methods for tracking messages and measuring their *impact* (i.e. effect on social interest) such as through the use of advertising value equivalents (AVEs, Macnamara, 2023). Meanwhile, in political science and media studies research, traditional “yardsticks” of newsworthiness and “social reach and impact” have tended to be equated with features such as the *selection, extent, duration*, and *half‐life* of news coverage, sometimes known as “issue attention cycles” (Binder et al., 2014; Wirz et al., 2021).

Historically, the tools and data available to many earlier *spotlight* risk amplification studies have been subject to practical constraints that impinged on access to news repositories and subsequently media corpus size and variety (Kasperson et al., 2012). Digitization, together with increased computer processing power, has newly afforded opportunities for the comprehensive investigation of risk news coverage (Chung, 2011; Jacobi et al., 2016; Krimsky, 2007; Wardman, 2017). This has included testing the applicability of computer‐assisted methods to corpus linguistics analyses of risk news coverage (Zinn & Müller, 2022), as well as identifying risk amplification trends, topics, and dynamics (Grundmann, 2021; Zinn & Müller, 2022). Studies in this vein have widely encompassed: collecting and analyzing historic media archives (Jacobi et al., 2016); examining news coverage of acute “events” (Wirz et al., 2018); tracking enduring “issues” (Hase et al., 2021); providing focused analyses of single risks (Rooke & Burgess, 2022); and making comparative observations derived from news coverage of multiple risks (Zinn, 2018). Building on these advances, and the contributions of earlier *spotlight* SARF studies, in the next sections we set out the methods employed in our *floodlight* approach, outlining the process for obtaining a relevant and comprehensive corpus of risk‐related news in US media, as well as differentiating categories of risk news subtopics and patterns of media coverage within these data.

## 3 METHODOLOGY

### 3.1 Determining a relevant news data set

In order to identify relevant media risk reporting, we collated a substantial corpus of risk‐related news articles between January 2000 to July 2021 from the *LexisNexis* database (see also Rossmann et al., 2018; and Friedman & Egolf, 2011 for use of this database). To ensure the high relevance of our corpus, we employed some refinements to our data search so as to exclude articles at the margins of risk reporting. Particularly, we screened the *LexisNexis* articles for the use of risk‐related keywords obtained from the 2018 SRA Glossary of risk terms.^1^ We selected keywords from this glossary because it provides “well‐defined and universally understood terms and concepts” with respect to risk (SRA Glossary, p. 1) and could therefore be employed to collate articles from across a spectrum of different possible risk domains. For SRA Glossary terms that had dual meanings in English‐language usage, we removed terms where the primary meaning, according to Merriam‐Webster, is not risk‐related. This left a refined set of risk‐related keyword search terms: *risk; uncertainty; harm; damage; hazard; safe; safety; security; secure; threat; resilience; vulnerability*. To further determine a news article as being sufficiently risk‐focused for final inclusion in our database, we also required at least one mention of the term *risk*, as well as the mention of at least one additional risk‐related keyword search term in the article text.

The initial search results from *LexisNexis* contained 520,374 print and online news articles. After filtering out close duplicates (a common feature of news databases due to syndication) along with other standard verification checks, we obtained a corpus of 283,165 risk‐related news articles.^2^, ^3^

### 3.2 Topic modeling—Processing textual data

We adopted a topic modeling approach to identify the common topics in our news database, that is, the topics that best explain the commonalities between clusters of risk news coverage. To identify the main overarching themes and patterns of risk news coverage, our topic modeling approach first employed natural language processing (NLP) techniques to convert the content of our news articles into an analyzable format (Hunt et al., 2020). In this initial step, we identified the relevant part of each news article that referred to risk. We chose this over using the full article in order to reduce the amount of nonrisk discussion of news stories included in our final text data set. For each article, we retained the sentences within that article that contained a risk term as well as the sentences before and after that key sentence. This allowed us to retain both the risk‐relevant term usage as well as contextual discussion around it.

The second step involved working in a *Python* coding environment, whereby each news article was tokenized, or separated, into individual words and other writing features such as numbers and punctuation. We removed text features such as punctuation, uppercase letters, extra white spaces in the text, numbers, and special characters from these individual terms. This also included high‐frequency terms known as “stop words,” which are functional words used so frequently so as not to be analytically informative to context and meaning. For this, we used the *Python* stopwords lists from *Natural Language Toolkit (NLTK)*, and *spaCy*, which includes common stop words such as “*and*,” “*of*,” “*the*.”

In step three, we adjusted the remaining words in our data set by converting them to the lemma (the dictionary base) of the word. We used a word's lemma to match different forms of words to their common lemma. For example, using this approach, the words finance, finances, and financial would all be mapped to a single word: finance. To implement this, we employed the *Python* package: *spaCy Lemmatizer*.^4^ We also inspected the remaining terms to see if any needed to be manually adjusted. This included the most frequent terms and manual removal of terms that are unrelated to risk or risk discussions, such as the day of the week or other time signifiers. This manual inspection is labor‐intensive, but significantly improves the quality of the data analysis (Aggarwal & Zhai, 2012).

In step four, we prepared the remaining terms for testing. We generated a Term Document Frequency matrix, which represents news articles (documents) in matrix form in which the rows correspond to all terms across all documents, columns correspond to all documents in the corpus, and cells correspond to the weights of the terms per document. We adopted the conventional choice, which is to consider one‐word (one‐gram), or two consecutive words (bigrams) terms. After building the corpus of terms, we found some risk‐related words (an obvious example being the term *risk*, which, because of the search process is required to be in each article) to be present in a large number of articles, as well as a long tail of rarely used terms. We therefore excluded terms that appear in more than 10% of all news because these words are too common to be useful in delineating risk subtopics. We also excluded terms that appear less than 10 times because these words are too rare to be useful. The latter adjustment removed most bigrams as bigrams are, by their construction, combinations of terms.

#### 3.2.1 Identifying risk topics with topic modeling

Topics in our news data set were identified using a latent Dirichlet allocation (LDA) model (Blei, 2012; Blei et al., 2003). We applied the LDA implementation of the *Python Gensim* package alongside the *Mallet* package (McCallum, 2002). *Mallet* has an efficient implementation of the LDA which is known to give clear topic segregation. LDA starts with a base assumption that the words chosen by the writer reflect the topics they wish to write about. By inspecting the observable words, word co‐occurrences, and word similarities across related documents, we can therefore uncover the topics which are hidden (or “latent”). LDA uses a probabilistic technique based on Dirichlet priors and a machine‐learning based expectation‐maximization algorithm to identify these hidden topics across documents. A key advantage of LDA over earlier similar methods, such as latent semantic analysis (LSA), is that the topic identification in LDA takes account of context. While LSA relies heavily on keyword identification, LDA determines topics based on the co‐occurrence of a range of terms. This means, for example, that the occurrence of a single term in a document is unlikely to identify a document as belonging to a topic if there are a range of other terms that indicate a document best belongs to another topic (Chang et al., 2009). This attribute is clearly useful in our *floodlight* approach as we want to be sure of the correct risk context of a news article—something that could be missed with a *spotlight* approach.

It is not known in advance if, for example, a corpus (in our case 283k news articles) is best described as having 10, 20, 50, shared topics across the documents in the corpus. The approach to solving this issue is to test a range of models with differing numbers of topics and then use the model‐outputted coherence scores, as well as manual inspection of the topics, to determine the best number of topics specification (Boyd‐Graber et al., 2017). Following this approach, we tested across a range of potential total number of topics of between 5 and 55, and concluded that a 35‐topic model best represented our data.

The output from LDA for each topic is a topic number and a list of terms that best describe that topic. Each term has a *beta* value that quantifies its importance to the topic. To assign descriptive labels to each topic, two researchers independently coded each topic based on the highest beta terms per topic with reference to a corresponding random sample of 20 news articles pertaining to that topic to ensure coherence. Each topic label was agreed upon following standard methodological protocols common in data‐driven thematic analysis (Braun & Clarke, 2006).

**FIGURE 1:**
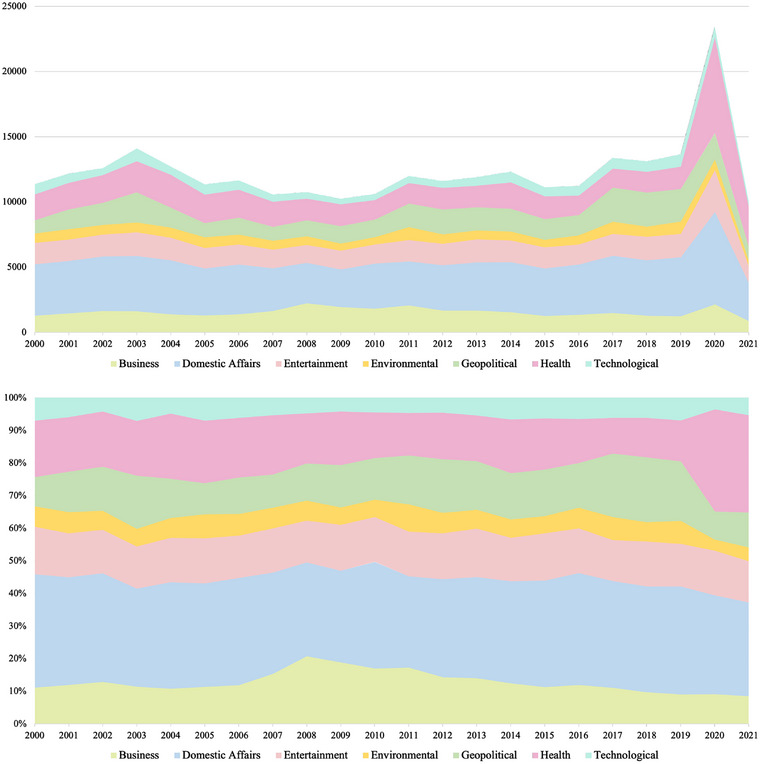
Distribution of risk category coverage in US media over time. Top figure: news article count per year. Bottom figure: news article count per year normalized by total articles per year.

After labeling, we excluded three topics along with their associated news articles from further analysis as they were considered to have very low relevance, which can be a common feature of topic modeling (Chang et al., 2009). Subsequently, our total corpus of news articles fell from 283,165 articles after initial filtering, to a final data set of 271,854 articles. The final 32 topics derived from our LDA analysis provide the main basis for analysis,^5^ we consolidated these topics into seven broad categories of US risk news following thematic coding protocols. The underpinning first‐order topics labels (determined by LDA) were used to develop consensus on labeling the thematic categories of risk news. The composition of the second‐order categories and first‐order topics are reported in Table 1, with Figure 1 showing the distribution of the category themes over time.

**TABLE 1:** Categories of US risk news (2000–2021).

	Business	Domestic affairs	Entertainment	Environment	Geopolitics	Health	Technology
*Panel A: Top contributing topic terms toward each category*							
1	investment (7%)	home (2%)	life (5%)	water (10%)	war (6%)	disease (5%)	technology (5%)
2	investor (7%)	law (2%)	play (5%)	climate_change (5%)	force (4%)	doctor (4%)	datum (5%)
3	business (5%)	issue (2%)	injury (5%)	region (5%)	attack (4%)	health (4%)	information (5%)
4	money (5%)	lawyer (1%)	game (5%)	plant (5%)	troop (4%)	patient (4%)	system (5%)
5	credit (4%)	lawsuit (1%)	sport (5%)	damage (5%)	Iraq (4%)	woman (4%)	internet (5%)
6	deal (4%)	prison (1%)	season (5%)	Florida (5%)	Israel (4%)	product (3%)	network (5%)
7	bank (4%)	charge (1%)	team (5%)	storm (5%)	soldier (4%)	virus (2%)	computer (5%)
8	firm (4%)	court (1%)	league (5%)	home (5%)	security (4%)	vaccine (2%)	access (5%)
9	loan (4%)	judge (1%)	fan (5%)	disaster (5%)	army (4%)	coronavirus (2%)	security (5%)
10	sale (4%)	attorney (1%)	player (5%)	area (5%)	Afghanistan (4%)	disease_control (2%)	service (5%)
11	cost (3%)	trail (1%)	field (5%)	site (5%)	agreement (2%)	infection (2%)	damage (3%)
12	stock (3%)	jail (1%)	show (4%)	chemical (5%)	Russia (2%)	mask (2%)	ship (3%)
13	economy (3%)	election (1%)	character (4%)	level (5%)	Syria (2%)	prevention (2%)	mission (3%)
14	market (3%)	party (1%)	story (4%)	exposure (5%)	Iran (2%)	study (2%)	space (3%)
15	growth (3%)	vote (1%)	movie (4%)	air (5%)	region (2%)	cancer (2%)	line (3%)

*Panel B: Distribution of seven categories across all news articles*							
%	13%	31%	14%	6%	13%	17%	6%
*Panel C: Distribution of 32 individual topics across each category*							
	Fin markets	Air safety	Human int	Pollution	War	Female health	R&D
	Fin management	Crime	Arts	Nat disasters	Conflict	Food safety	Cyber sec
	Banking	Locality	Sports		European affairs	Illness	Tech failure
	Job security	Compliance			Trade	Medicine	
		Child welfare				Infect disease	
		Education					
		Road safety					
		Party politics					
		Policing					
		Regulation					
		Elections					

*Note*: This table presents the seven second‐order categories of US risk news constructed from 32 individual first‐order topics. The individual topics are reported in the Supporting Information, Tables S3 and S4. Reported for each category are three panels:

‐ **Panel A** reports the top 15 terms that most contribute to topics in that category. The percentage (%) next to each term indicates the proportion of articles in which that term is prominently featured within the respective category.

‐ **Panel B** presents the distribution of the seven second‐order categories across all news articles (total 271,854 articles).

‐ **Panel C** lists the individual first‐order topics that contribute to each second‐order category.

### 3.3 Distinguishing signal events from risk news

Given our *floodlight* approach, we are interested in the relationship between general risk news coverage *within* a risk category of signal events that emerge as particularly prominent within that category, and also signal events that are prominent when all risk news is considered *between* all categories. Our first step to distinguish signal events *within* a risk category is to de‐noise the time series of risk news for each category. For this, we use locally estimated scatterplot smoothing (LOESS) to smooth the time series by accounting for seasonality and time trends (Cleveland et al., 1990). A similar smoothing approach has been adopted in recent studies of Russian‐influenced news coverage in Ukraine (Watanabe, 2017) and public discourse around measles outbreaks (Zhao et al., 2020). After controlling for seasonality and time trends,^6^ the smoothed time series is then tested for anomalously high news coverage days as *potential signal event* days due to their indication of a sudden growth in news coverage. The testing method, the generalized extreme studentized deviate (GESD) test, is well‐established for identifying anomalies in smoothed time series of news, including COVID‐19 social media news coverage (Yousefinaghani et al., 2021).

Smoothing and then GESD testing are performed for each category, the test output provides a list of days per category time series with anomalous news coverage. From this point forward, we concentrate on these days as *potential signal event* days. Across the seven‐category time series, 237 potential signal event days were identified at this stage. That is, the news coverage on these days, for a category, is anomalously high.

Some further stages are necessary for determining prominent signal events *within* risk categories. Given that sustained *share of the ink* is considered to be reflective of heightened media attention (Kreps, 2014; Wardman, 2017; White, 1950; Vasterman, 2005), we first focus on whether a signal event day occurs within a cluster of anomalous news days as a core indicator of continued media amplification. In order to further classify emergent news as a signal event, we then specify that there must be at least two anomalously high volume days relative to all risk news within a 30‐day period in a category. By reading the underlying news stories that are related to those two+ days, we ascertained if there was a common link between the days in terms of risk news focus. Lastly, to remove potential events in low news frequency categories,^7^ we required that there should be a minimum of 15 news stories on each day related to the core signal event. The confirmed events elicited by this process reflected the emergence of a signal event that equated to at least one risk news story, per day, per media outlet in our data set, thus indicating a reasonable degree of widespread coverage within our subset of 17 news outlets. In order to identify the prominence of signal events *between* risk categories, we repeat the same steps outlined above without disaggregating our data according to each risk category, therefore allowing for all risk news, across all categories, to be considered relative to each other.

## 4 FINDINGS

### 4.1 US risk news overview: Key terms and categories

From our data, 32 risk news topics were extracted and thematically grouped into seven overarching risk categories named: *business*, *domestic affairs*, *entertainment*, *environment*, *geopolitics*, *health*, and *technology*.

As shown by the thematic grouping in Table 1, along with the visual timeline of each category in Figure 1, it can be seen that *domestic affairs* is the largest risk category for this period accounting for 32% of risk‐related news overall, almost twice that of any other category. *Health* is the next largest category accounting for 17%, with *entertainment* 14%, *geopolitics* 13%, and *business* 12%. The remaining two categories, *environment* 6% and *technology* 6%, are the smallest categories of risk news in our data set.^8^

**FIGURE 2:**
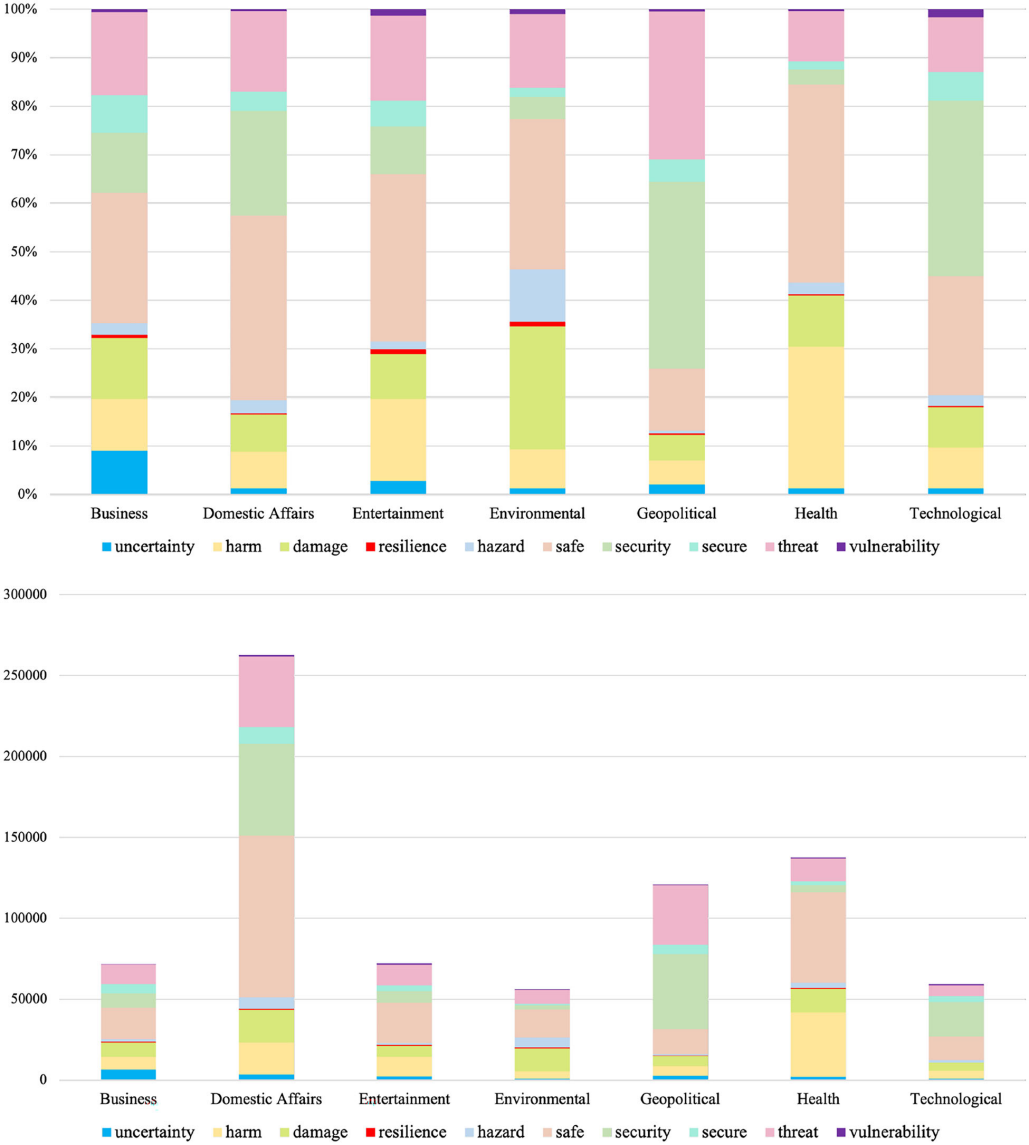
The Society for Risk Analysis (SRA) 2018 (reduced) glossary terms propensity. The top figure is a stacked count of term occurrences. The bottom figure is a 100% stacked chart. The term *risk* is removed from these charts, as it is required to be present in all articles as per the search criteria.

Some terminological features of risk reporting in US media over this period are evident from Figure 2. After the word *risk*, the three most frequently used SRA Risk Glossary terms were *safe*/*safety*,^9^ *security*, and *threat*, whereas the least utilized terms are *resilience* and *vulnerability*. While all the SRA Glossary terms tend to appear in all categories, there is also notable variability in the prominent use of certain risk‐related terms' words within each news category domain. We observe, for example, that the geopolitical risk category predominately makes use of the terms *threat* and *security*, whereas, in addition to these terms, the domestic affairs category also largely employs *safe*/*safety*. The health category predominately uses the terms *harm* and *safe*/*safety*. In contrast, the environment category makes more use of the terms *hazard(s)*, *damage*, and *resilience*. The two standout terms for the technology risk category are *security* and *vulnerability*, while business risk makes most use of the term *uncertainty*. Lastly, by contrast, the share of words in the entertainment category is fairly evenly distributed with *resilience* and *vulnerability* figuring most prominently for this risk.

### 4.2 Risk amplification: Signal events, trends, and patterns

**FIGURE 3:**
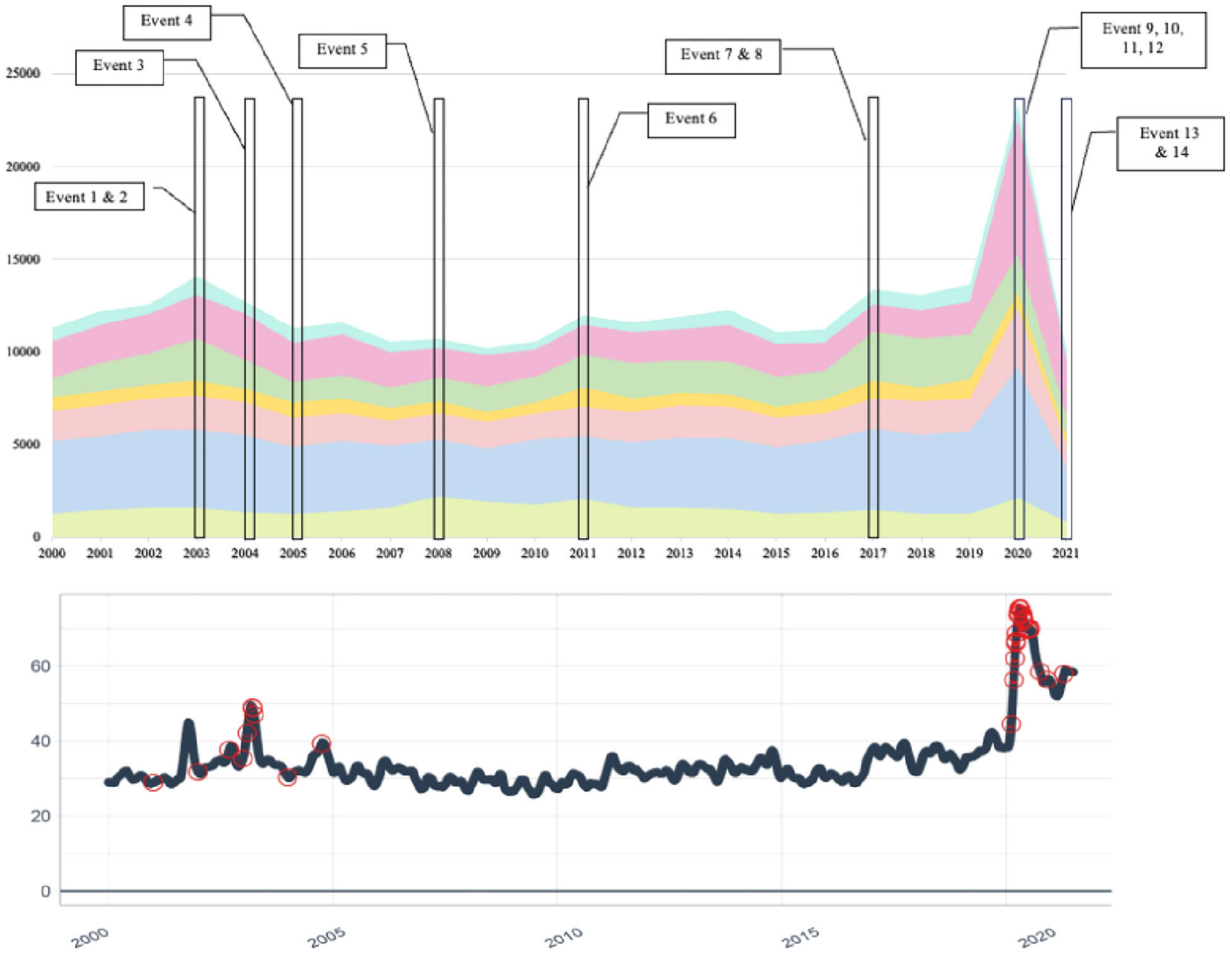
Top figure shows the 14 signal events identified across the timeline *within* categories. Event 1 is the Columbia shuttle disaster; Event 2 is the Iraq war. Event 3 is the Vioxx drug; Event 4 is Hurricane Katrina; Event 5 is the financial crisis; Event 6 is the Fukushima disaster; Event 7 is the US–Asia relations; Event 8 is hurricane season; Events 9, 10, 11, and 12 are the COVID‐19 and its associated impacts; Event 13 is the US withdraw from Afghanistan; Event 14 is the COVID‐19 vaccine. The methodology for identification of the signal events is described in Section 3.3, and further details on each event are summarized in Table S2. The bottom figure shows trend and signal events that are significant “*between*” categories in the overall timeline.

Our analysis identified 14 signal events *within* the seven categories in our news media timeline chronicled in Figure 3.^10^ The *health* category has the highest number of signal events (N=4), followed by geopolitics (N=3), and environment (N=3). All other categories contained one signal event. Overall, these 14 signal events are associated with about 3% of the news articles in our data set,^11^ with each event receiving an average of 40 days of news coverage. There was considerable variance in these news reporting cycles per event with, for example, Event 10 having a 169‐day reporting cycle, and Event 7 having just a 2‐day news cycle. This overview indicates that certain categories may be more prone to signal events than others, and reported for a longer duration, although signal events do occur in all areas of risk news.

Looking at media reporting for the signal events identified, some key features are evident. For example, as shown in Figure 3, the 2003 Columbia space shuttle disaster (Event 1) occurs within the *technology* category, with media amplification for this event clearly observed through the large spike in news coverage in the timeline. Sample news article text for this event also shows successive media reports highlighting the importance of “newsworthy” issues such as “poor risk assessment” by NASA (see Table S2). The pattern of media amplification *within* the technology risk category indicates a general trend of signal event‐driven news, even if not all become signal events by comparison. One example is a problematic NASA space shuttle flight in 2005. While not resulting in the same level of news coverage as the Columbia shuttle disaster, this signal event does suggest heightened sensitivity within the media to these types of risk problems and the reasons they occur. By contrast, the pattern of news attention in the later period of *technology* risk news exhibits a smoother profile. Here, media focus was more evenly distributed across a range of issues such as newly emerging technologies and how their associated threats have become embedded within the everyday lives of US citizens. More moderate levels of amplification are accordingly demonstrated through news coverage associated with issues such as cybersecurity and a series of publicly documented data breaches including in well‐known companies such as Target, Yahoo!, Equifax, and Sony.

Similar observations can also be made regarding the contributions of other signal events *within* their respective risk categories amidst the general ebb and flow of their risk news. In particular, signal events tend to generate new attention within a category. For example, in the *domestic affairs* category, increases in media reporting regarding *air safety* followed the 9/11 terrorist attacks. The global financial crisis (Event 5) figured as a key risk event in the *business* category and was associated with the collapse of major investment banks such as Lehman Brothers and Bear Stearns, along with a US government scheme (Troubled Asset Relief Program) to stabilize the banking sector. This heightened attention and focus then turned to the subsequent domestic and global economic recession.

The *environment* risk category consistently received some of the lowest levels of news coverage overall in our time series. This category did however feature three signal events relating to the Fukushima disaster (Event 6), Hurricane Katrina (Event 4), and the disastrous hurricane season in 2017 (Event 8). There is a distinct possibility that the amplification of these events was because of the “suitability” of extreme nature events for 24/7 news channels and the subsequent generation of striking visuals for news stories.

The two main risk topics within this category were *pollution* and *environmental disasters*, which also reflected acute industrial disasters affecting the environment (e.g., Deepwater Horizon) as well as the ongoing issue of climate change. Among signal events featuring in the *geopolitics* category were tensions between the United States and North Korea (Event 7) primarily concerning threats posed by new weapons capabilities. These issues started to appear with the presidential election of Donald Trump and a subsequent trade war with China (as seen in the expansion of the *trade* topic over the 2017–2020 time period). The US withdrawal from Afghanistan (Event 13) also figured as a major instance of media amplification. Lastly, in the *health* category we can see high levels of media amplification regarding the 2004 withdrawal of the Vioxx drug from the market due to severe side effects (Event 3), as well as reporting surrounding COVID‐19. Other signal events figure in the timeline, including the outbreak of H1N1 (swine flu) in 2009 and the emergence of the Ebola virus in 2014, but these did not achieve the same levels of media amplification by comparison.

### 4.3 Cascading risk amplification, compound events, and attenuation

When we examine the overall news timeline and consider the prominence of signal events *between* categories, our signal events analysis points to two highly distinct cases of *cascading* risk amplification (see Figure 3) compared to the other 12 signal events. In both cases, a series of interconnecting compound signal events can be observed. The first case in the timeline concerns the geopolitical incidents associated with the “War on Terror,” while the second concerns the “COVID‐19 pandemic.”

The significance of the combined events labeled by the US domestic media as the “War on Terror” is indicated first by the scale of anomalous news coverage toward the beginning of our timeline. A marked shift in growth is reflected at a holistic level in the overall news timeline seen in Figure 3, but at the more granular level it can be seen that this is driven by aggregate news media attention that originated in the *domestic affairs* news category, with a 290% growth in media reporting around “Air Safety” in the period following 9/11, that then transcended and dominated the *geopolitical* category for a sustained period of time.^12^ To quantify this, geopolitical risk news grew 126% during the period 2000–2003, a trend witnessed by no other risk category during the same time period.

Accounting for this growth, news coverage within the *geopolitical* risk category can be seen to reflect a series of compound signal events wherein media amplification encompasses reports of multiple, successive, interconnected, and high‐impact incidents. The predominant focus of geopolitical news coverage concerns heightened tensions regarding “*attacks*,” “*security*,” “*conflict*,” and *“war”* along with the seriousness of their consequences. For example, the news cycle regarding the Iraq War (Event 2) accounted for 30% of all reported news in that time period, highlighting issues such as the likely war casualties and resulting political ramifications. Text from one sample news article illustrates this^13^: *“For the first time since taking office, President Bush faces the possibility that large numbers of American troops might die on his watch, and his aides are scrambling to prepare the public for a toll that could rapidly erode its support for the war.”* The period 2000–2003 which encompasses 9/11 and the subsequent Iraq and Afghanistan wars saw a 224% growth in US risk news related to “*war*,” and a 175% growth related to “*defence*” reflecting the step change in risk news reporting narrative.

Patterns of cascading risk amplification associated with the “COVID‐19 pandemic” are in turn observed toward the end of our media timeline, and in certain respects can also be seen to display characteristics comparable to reporting of the “War on Terror” in terms of a series of prominent interconnected signal events. However, our analysis also indicates that COVID‐19 had a wider systemic influence, with overall levels of risk news increasing by 72% across all categories within US news media. This trend starts with elevated levels of media coverage associated with the COVID‐19 outbreak within the *health* category, which saw a 2368% growth in news articles in the outbreak period. This then leads to notable impacts across other categories within our timeline.

As with the “War on Terror,” it can be observed that COVID‐19 news reports follow a series of interrelated compound signal events which contribute to creating a case of cascading media amplification. Coverage of the pandemic initially begins with reports of the outbreak of COVID‐19 (Event 9, Table S2), then follows attempts to prevent the spread of the disease (Event 12, Table S2), before finally culminating with the launch of vaccines and issues of hesitancy surrounding their uptake (Event 14, Table S2).

Across these signal events, we see also the “risk message” notably changing. Initially, COVID‐19 news articles warned the public to prepare for the impending arrival of the virus, as illustrated by the *New York Times* in February 2020 “*Countries across the world may now be faced with the task of limiting the spread of the disease on their own soil*” (Event 9, Table S2). Eventually, this message changed to address the perceived risks associated with vaccine hesitancy illustrated in April 2021 “*the benefits of the Covid vaccines far outweigh the risks*” (Event 14, Table S2).

Elsewhere within the *health* category, news coverage illustrates the wider impacts of COVID‐19 reporting with regard to media attention to other health issues. For instance, the health timeline,^14^ shows “*food safety*” to be the only topic aside from “*infectious disease*” to see a notable increase in coverage during this period, with a rise of 243% in 2020. However, this increase primarily relates to news reporting around the safety of food establishments in light of COVID‐19, thereby indicating a spillover of COVID‐19 into this news category. Our *floodlight* also captured the attenuating effect of the pandemic on other health topics such as the ongoing opioid crisis (captured in the *medicine* topic) and *women's health*, which both saw less attention as COVID‐19 emerged as a prominent newsworthy story. This phenomenon is not typically observed using the *spotlight* approach to SARF given the inherent focus only on the event/incident under investigation.

**FIGURE 4:**
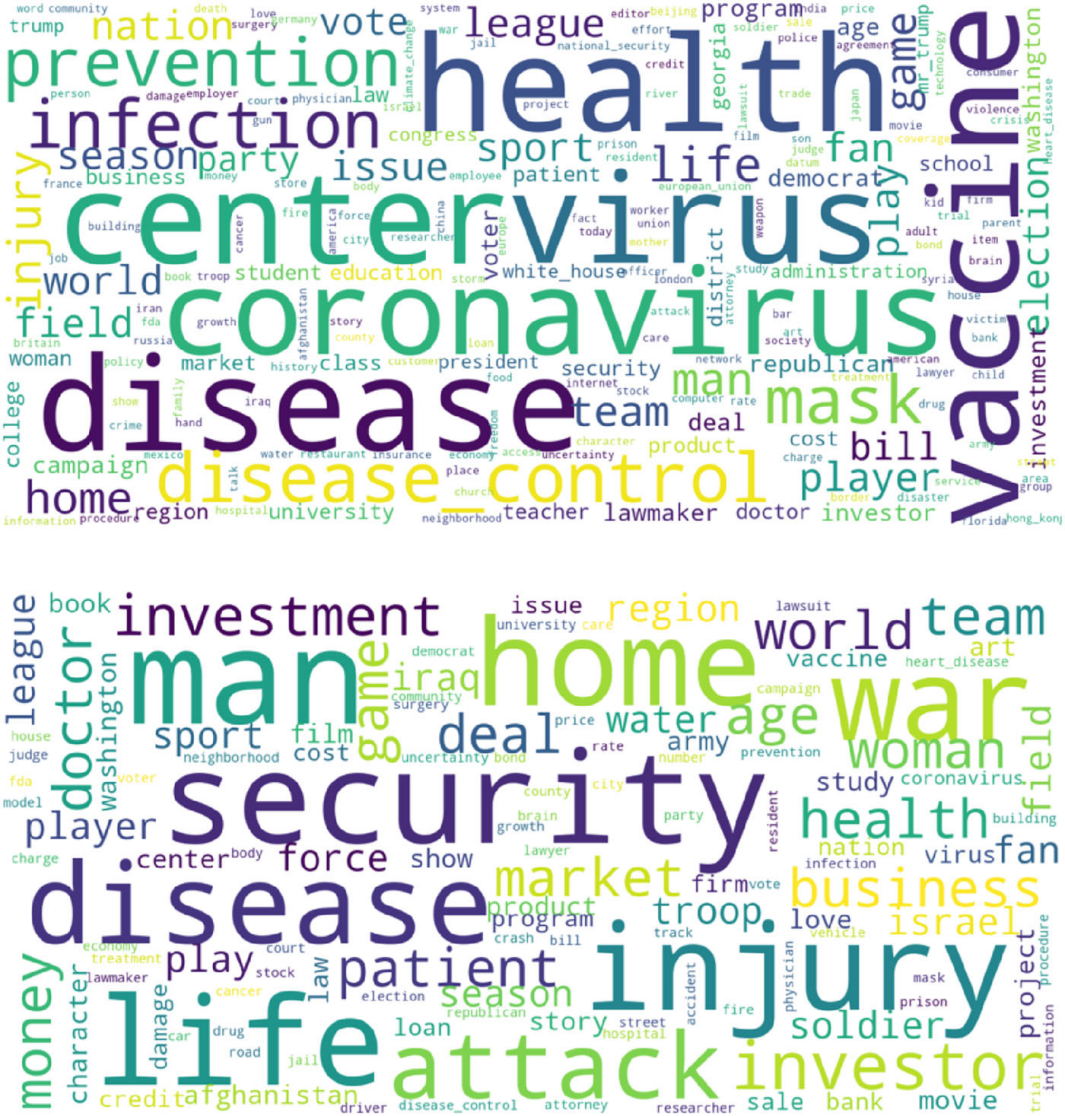
Word clouds: (Top) most popular terms in US risk news coverage for 2020 (COVID‐19 outbreak year), (Bottom) most popular terms in US risk news coverage for the full‐time period (2000–2021).

More broadly, coverage across all other categories similarly experienced the spillover of COVID‐19‐related news reports as the wider impacts of the crisis were felt around society. A comparison between the risks terms used in the overall time period and the risk terms used during 2020 is provided in wordcloud form in Figure 4. This shows that during 2020 there was a focal preoccupation with COVID‐19 with a heavy emphasis within news items on infectious disease or the consequences of the disease. Notable growth was also witnessed within news categories for issues not typically related to disease or health, such as *job security* (due to temporary unemployment) which grew 255%, and *child education* (due to the closing of schools) which grew 216%. These increases reflect the significant impact of lockdowns as a means of bringing the disease under control, along with their wider effects which led to distinct signal events in the categories of domestic affairs and education, respectively.

The only signal event in the *entertainment* category was also COVID‐19‐related (Event 10, Table S2). This event specifically concerned the impact of COVID‐19 on both the safety aspects and curtailing of sporting and entertainment activities, respectively. Up until this point, news stories over the time period for the *entertainment* category were more focused on issues such as sports player injuries and risk‐related decisions of players and teams on the sports field. The *domestic affairs* category was also likewise monopolized by COVID‐19 news in 2020 relating to the “everyday life” impact of the pandemic (Event 11, Table S2), albeit a notable increase in the *policing* topic in 2020 regarding the *Black Lives Matter* movement can also be discerned. For *business*, whereas previously the *banking* topic had been a driver of events such as the global financial crisis, the impacts of COVID‐19 clearly took precedent regarding news issues such as the impact of the pandemic on the viability of businesses, security of jobs, and the US economy.^15^

## 5 DISCUSSION

The primary objective of this study was to determine prevalent patterns of risk amplification and attenuation within US news media regarding how media attention varies across different risk topics and signal events, respectively, over time. A holistic analysis was facilitated through the development of an innovative inclusive *floodlight* approach that incorporated risk‐related search terms drawn from the SRA Glossary to generate a large corpus of US risk‐focused news articles over a 20‐year period. The use of a general set of risk‐related terms allowed for a broader examination of our corpus of risk news than would otherwise have been possible had we taken a conventional *spotlight* approach that simply collated articles using keywords relating specifically to preselected events/incidents/risks. Subsequently, we were able to delineate general categories of risk news, and the topics that comprised them, as well as uncover historic patterns and signal events reported underneath them that stood out relative to all US risk news over the time period. Specifically, our analysis resulted in the identification of seven risk news categories and 14 risk amplification signal events from which the novel occurrence of two distinct cases of cascading media amplification was discovered relating to the “War on Terror” and the “COVID‐19 pandemic.” These findings thus serve to provide a broad backdrop against which to contextualize previous risk amplification studies that have gathered news in relation to a specific risk event or topic in isolation, or employed a much narrower range of news sources. Some key aspects of our analysis and findings warrant further reflection and discussion, which we now address in turn.

First, our study demonstrates that US media attention to risk is not merely concerned with extraordinary events and crises. Our findings also support earlier work showing that media coverage is permeated by risk discussion that widely reflects a cross section of different segments and features of everyday life (Hamilton et al., 2007; Vasterman, 2005; White, 1950). This is demonstrated by the large variety of risk topics extracted from our corpus and the variability of different news categories. What is more, the prevalence of certain risk‐related terms evidenced between categories also indicates not only that risk is reported in different ways, but that there is an internal coherence to how risk is framed and understood within specific news domains. Therefore, while risk reporting might be said to attend to the most salient features of a particular risk issue or event, it can also be observed to follow reporting conventions in which there are predominant linguistic patterns in the choice of terms used by journalists when writing about risk within different domains of interest. These linguistic patterns might mirror the way in which risk is often conceptualized and defined differently according to the orientation and focus of different scientific disciplines (Aven, 2012). Nonetheless, it raises questions regarding, first, whether journalists and editors working in one area (e.g., *health*) understand and make sense of risk differently from those in other areas (e.g., *business*); and second, what impacts this might have on audiences. For example, do those who are interested in writing and reading about *technology* news come to understand “risk” predominately in terms of security and vulnerability, while those who are interested in *health* come to understand risk in terms of harm and safety? It may be that people's conceptual understandings of “risk” are framed by their “news diet” in this way, or that some are adept at interchanging risk concepts depending on the context or story of interest. Indeed, do those who have a broader diet of news, for example, that includes *health, environment*, and *entertainment* news, have a broader conceptual repertoire for understanding risk in more nuanced ways than those who are just simply interested in reading the *business* pages? Whatever the case, it underscores the value of the SRA Glossary in providing a comprehensive lexicon of widely applicable risk‐related terms and concepts for obtaining risk‐related news appropriate to different domains and reader interests. By extension, the glossary may be of broader utility not only for risk analysts but also for risk science communicators attempting to report on risk in ways that resonate well with interested readers.

Second, while a variety of risk news categories were observed, it is notable that *domestic affairs* was the most prominent in that it obtained the highest level of news coverage both in terms of article count per year and the normalized percentage of articles across all of the years in our time series. This predominant focus within our corpus might naturally reflect the US basis of the news sample we obtained, for which media attention is likely to be intrinsically biased toward reporting domestic issues and events of regional and national interest. There are also some other possible contributory factors to consider. For instance, the *domestic affairs* category incorporates a comparatively high number of topics (n=11) that are also wide‐ranging in scope from *air traffic safety* through to *crime* and *policing*, as well as *education* and *elections*. While our linguistic analysis shows that these topics largely pertain to threats to security and safety, it is apparent that inherent variety is a distinctive feature of domestic risk news. Consequently, this wide focus means that there is a high chance of a risk‐related incident (in one guise or another) being reported in the *domestic affairs* category on any given day. In our time series, the *domestic affairs* risk category accordingly only dipped below 3000 articles per year once in 2009.

The *domestic affairs* finding stands in contrast to the *environmental* risk category which received a much smaller amount of media attention. This is clearly illustrated by both there being only three signal events and that the category achieved over 1000 articles annually only once in 2011. Reasons for less media attention in this category could reflect, on the one hand, the inherent features and characteristics of environmental risk. For example, while major environmental disasters are typically considered significant newsworthy events—as evidenced by coverage in our data relating to Deepwater Horizon and Fukushima—historically they have tended to be relatively sporadic in nature. It may also be the case that despite having grown in prominence, environmental issues such as climate change have comparatively fewer events reported on a day‐to‐day basis due to the low frequency and magnitude of individual impacts that might otherwise generate headline news. On the other hand, the relatively low levels of attention observed might have a cultural basis. As recent work by Newman et al. (2022) shows, only 30% of US readers are interested in climate change‐related news, perhaps reflecting the general finding that natural hazards tend to evoke less concern than technological hazards (Kasperson et al., 1988; Paté‐Cornell et al., 2018). Our linguistic analysis also identifies that risks in the *environmental* category are described in conjunction with terms such as hazard, damage, and resilience, which may be of less personal consequence to readers than more dreaded characteristics such as those affecting personal harm and safety. This pattern of reporting may change over time as the urgency of the current climate crisis and its effect on more arenas of everyday life becomes more evident and thus a more newsworthy domestic issue meriting media attention. Subsequently, further efforts may be needed to equip journalists and editors working across different news desks with the knowledge required to report environmental issues and complexities for their readers (Newman et al., 2022).

Third, our signal events analysis was able to confirm the novel occurrence of two distinct cases of unparalleled cascading media risk amplification underpinned by compound signal events that occurred “*between*” categories in our time series. In the case of the “War on Terror,” media attention was initially spurred by 9/11 and the subcategory of airline safety before subsequently encompassing geopolitical tensions associated with reporting different aspects of the Iraq War. Similarly, for COVID‐19, we initially observed a high level of news media reporting within the *health* category, but in addition, we saw news coverage continue to display dynamic movement across different categories of “nonhealth” risk news as key events associated with the crisis unfolded in keeping with prior findings (Wirz et al., 2021). For other risk events, examples of the transferal of risk information and signals around society can be found (Binder et al., 2014; Kasperson et al., 1988; Rossmann et al., 2018; Vasterman, 2005; Vasterman et al., 2005), but to a much lesser degree as reporting of these events tends to be comparatively localized within particular news categories.

We suggest that the marked differences observed in our study regarding these two cases could be related to the magnitude of ripple effects associated with the compound nature of the risks in question. In this way, both crises presented editors and journalists, the “gatekeepers” of news, with an enduring newsworthy story that pervaded across business, social, policy, and entertainment arenas (Binder et al., 2014; Frewer et al., 2002; Jagiello & Hills, 2018; McInerney et al., 2004; Rossmann et al., 2018; Wong & Yang, 2022; Young et al., 2008). This is clearly displayed in Figure 4, for example, where the issue of COVID‐19 dominates all other news in 2020. Under ordinary circumstances, *Entertainment* and sports news journalists would not typically report a novel health issue for its own sake, rather it would have to have had a direct impact on issues and events of interest to that news category. Likewise, *business* news editors would not be specifically interested in COVID‐19 except where it pertained to core business interests, such as the closure of businesses and heightened unemployment, the rise of remote working, and the benefits to pharmaceutical companies of developing a new vaccine.

Rather, the advent of the “War on Terror” and “COVID‐19 pandemic,” respectively, accordingly led to cascading risk amplification due to a series of compound signal events whose consequences were direct, severe, immediate, long‐lasting, and far‐reaching (Baker et al., 2020; Chen et al., 2020). In our view, this led to two distinctive situations where the “issue attention cycle” of each reflected both Stage 1 amplification through prominent coverage of a particular signal event relative to all risk news (this relativity is an important addition of the “*floodlight*” approach), as well as reporting on widespread Stage 2 ripple effects that impacted multiple aspects of US society as seen, for example, by Events 9, 10, 11, 12, and 14, and Table S2 (in Supporting Information). This finding can thus be considered as distinct from the more well‐established case of “secondary amplification” whereby multiple parties act as amplification stations whether all simply reporting on the same event or to purposely promote an issue of concern (Kasperson et al., 1988; Wardman & Löfstedt, 2018).

One further finding of note is that a novel form of attenuation of other risk issues and events was also apparent. In this study, risk attenuation occurred not simply due to declining interest per se, but through a “crowding out” effect whereby more regular health risk news was eclipsed by a more prominent news story. In this case, the crowding out effect was observed particularly around the topics of female health and medicine safety (e.g., opioids, Cantor et al., 2022). This phenomenon has been noted in news media discussions of regarding the impact of major events as well as the knock‐on impacts of COVID‐19 (Lakhani & Glenza, 2021), but our observation of an enduring attenuating effect has not yet been confirmed in the academic literature until now. This finding therefore underscores another benefit of a *floodlight* approach over a *spotlight* approach for SARF research, whereby adding a systemic level of analysis helped to trace the relative waxing and waning prominence of risk reporting for different issues simultaneously as they competed against one another for media attention.

## 6 CONCLUSION

News media are incredibly important for generating awareness and understanding about risk, framing risk discussions, and providing a credible and trustworthy source of risk communication to help guide behavior. The majority of previous SARF research might be considered to have adopted a *spotlight* investigative approach to examine certain risk events, in a particular place, at a specific point in time. In this study, we examined risk amplification within US news media over the past two decades using a *floodlight* approach to map out and analyze at a macro level the holistic contours and dynamics of prominent reporting patterns and key signal events. This meant that is was also possible to identify widely varying types of amplification and attenuation processes that have taken place, which to date have not been considered by the studies employing the more common *spotlight* approach. As such, the ability to consider the social amplification of a risk event, relative to all other risk news is considered a key aspect of this *floodlight* approach. Furthermore, to our knowledge this study is the first to employ the SRA Risk Glossary, notably demonstrating the utility of using the SRA Risk Glossary to capture the wider context of media risk amplification than achieved previously.

Nonetheless, some cautions regarding the interpretation of our findings are also to be noted. First, as commented above, while incorporating a large number of news sources by conventional research standards, our corpus was confined to a sample of 17 national US news media outlets. This national focus potentially limits the generalizability of some of our key findings to other nations and to local news which may display different amplification characteristics. For instance, local news, which may vary from place to place, and incorporates a large part of the media that people access for information, particularly during crisis events that have regional impacts and implications. These considerations also extend to textual analyses using other languages. Further research is subsequently needed to see if our topics, categories, and signal events are replicated both in news around the globe and in local news, to address any regional differences. Second, due to space limitations, we were not able to provide a full in‐depth analysis of all seven categories of risk news, their associated topics, and signal events over the last 20 years. A “deep dive” into each of these, including performing additional sentiment analyses could be of further interest to multiple stakeholders in the SARF community.

Third, we did not include social media as a news source. Future research could therefore employ a multilevel modeling approach to investigate how social and traditional media reflect or deviate from each other regarding attention and reporting of different risk topics and categories, as well as for signal events and cascading coverage across media. Fourth, we did not test how this larger news ecosystem might impact on public perceptions of risk. Further consideration will need to be given to how new media environments intersect with traditional news to transform people's awareness and experiential understanding of risk through features such as the “multimodel” mixing of text, image, and sound in contemporary media production and sharing typically intended to better capture people's attention (Wardman, 2017). One future development of *floodlight* research in light of these considerations would be to incorporate tools for analyzing risk amplification and attenuation dynamics and processes across various mediums (e.g., text and/or video) along with people's involvement and influence when contributing, modifying, commenting on, and/or sharing risk news (Wardman, 2017). Similarly, journalism is currently undergoing transformations through the wider adoption of Artificial Intelligence (AI) technologies. On the one hand, AI promises to increase the visibility, reach, and efficiency of risk news dissemination to prospective readers, but on the other hand carries with it a greater risk of bias, which runs counter to traditional news media values for honesty, accuracy, and accountability (Wardman, 2017). These are important considerations in the context of growing concerns over the spread of misinformation during risk events (Krause et al., 2022).

In closing, we suggest our findings contribute some important insights into the reporting of historic risk events and issues but also underscore the potential for further refinement of SARF through the adoption of a more expansive research agenda addressing more variegated forms and patterns of risk amplification and attenuation, which can assist future research studies and risk communication policy debate and development.

## Supporting information

Table S1: Risk‐related news dataset description
Table S2: Major risk amplification events featured in US news media
Table S3: US media risk topics (2000‐2021)
Table S4: US media risk topics (2000‐2021) (contd.)
Figure S1: Timeline of business risk news.
Figure S2: Timeline of domestic affairs risk news.
Figure S3: Timeline of entertainment risk news.
Figure S4: Timeline of environmental risk news.
Figure S5: Timeline of technology risk news.
Figure S6: Timeline of geopolitical risk news.
Figure S7: Timeline of health risk news.

## Footnotes

1 The SRA Glossary is publicly available at: www.sra.org/wp‐content/uploads/2020/04/SRA‐Glossary‐FINAL.pdf. The glossary is not an exhaustive list of risk terms, however, its well‐defined and universally understood terms and concepts mean that it is not incident/event/topic‐specific, thus ensuring that we do not inadvertently narrow the focus of our initial corpus.

2 Subsequent data decisions explained later in the study further reduce this to the 271,854 articles mentioned in the abstract.

3 Table S1 summarizes the text search parameters.

4 spaCy package: https://spacy.io/api/lemmatizer.

5 The details on each of these topics are reported in the Tables S3 and S4.

6 Seasonality refers to predictable and recurring patterns in news coverage that occur at regular intervals (e.g., increased health news during the flu season). Adjusting for seasonality is crucial to prevent these regular patterns from being misinterpreted as anomalies or significant risk events. This ensures that any news coverage that emerges as prominent is truly indicative of an unusual occurrence, rather than routine seasonal variations. Time trends refer to long‐term shifts or trends in the data, such as increased risk news coverage over time, changes in media reporting styles, or evolving public interests over the years. By smoothing out these trends, we aim to isolate and highlight abrupt and significant deviations in news coverage, which are our primary interest, from these gradual changes. This is essential for accurately identifying and analyzing genuine risk events in the context of evolving media landscapes.

7 In essence, it is easier to be an “anomaly” in low news risk categories, such as technology, because the daily average news count for the category is very low.

8 The composition of topics corresponding to each category can be also seen in Figures S1– S7.

9
*Safe* incorporates the term *safety* due to our data‐processing rules, which reduces terms to their common core.

10 These categories are elaborated on further in Table S2.

11 Although direct comparison of news article counts is not appropriate given the strong upward time trend in our data set.

12 See Figure S6.

13 See Table S2 for more sample news across all categories.

14 See Figure S7.

15 For the interested reader, see Figure S1.
