# Supplementary material for: Media amplification under the floodlight: Contextualizing 20 years of US risk news
Source: Risk Anal. 2025 Feb 5;45(7):1940–56. doi: 10.1111/risa.17701 (PMC12396942; doi:10.1111/risa.17701)

Supplemental Materials

Table S1: Risk-related news dataset description

**Data source**		
LexisNexis: Major US Publications (print and online) of 17 notable newspapers, which make articles available in English, including New York Times, and Los Angeles Times. Daily News (New York), USA Today, The Baltimore Sun, The Philadelphia Enquirer, The Philadelphia Daily News, Tampa Bay Times, St-Louis Post-Dispatch, Pittsburgh Post Gazette, The Atlanta Journal-Constitution, The Jerusalem Post (Intl Ed), The Daily Oklahoman, The Tampa Tribune, Star Tribune, The Christian Science Monitor, The Hartford Courant.		
**Initial article identification parameters**		
Society for Risk Analysis 2018 keywords (selection): risk; uncertainty; harm; damage; hazard; safe; safety; security; secure; threat; resilience; vulnerability.		
**Article search refinement**		
1. At least one mention of ‘risk’ in the article, and;<br>2. Must contain one additional Society for Risk Analysis 2018 keyword		
**Other search information**		
Date range: Jan 2000 - July 2021<br>*Language*: English language only		
**Descriptive statistics**		
Time period	Annual average news (All)	Annual average news (Refined)
2000-2005	12,951	12,377
2006-2010	11,224	10,766
2011-2015	12,247	11,785
2016-2021	16,019	15,426
Total news articles	283,165	271,854

Table S2: Major risk amplification events featured in US news media

	Category	Risk amplification event	Key dates	Sample news article text
1	Technology	Columbia shuttle	02/02/2003 - 09/02/2003	"The Columbia is lost. There are no survivors. The crew of the shuttle Columbia did not return safely to Earth, yet we can pray they all are safely home. May God bless the grieving families"; "NASAS own safety panel, in a prescient prelude to Saturday's loss of the space shuttle Columbia, twice within the last year expressed a high level of concern that budget cuts, aging spacecraft, and an eroding safety net posed a threat to the shuttle fleet"; "For Texans awakened by the "boom-boom," it was the sound of the sky falling. For the clinical voice of NASA Mission Control, it was a "contingency." For Americans already grappling with a confluence of threatening events, the instinctive reaction was, "What next?" "; "Too much money, too much risk, too much death"
2	Geopolitics	Iraq war	07/03/2003 - 30/03/2003	"On the eve of the U.S.-led war against Iraq, world leaders made their last attempts to avert it"; "This war risks, according to internal U.N. estimates, up to 500,000 Iraqi civilians dead and wounded, and no one knows how many American troops will perish"; "Bush's strategists believe, the fall of the Soviet Union in 1991 and the rise of a new terrorist threat a decade later have created the need for new global arrangements"; "For the first time since taking office, President Bush faces the possibility that large numbers of American troops might die on his watch, and his aides are scrambling to prepare the public for a toll that could rapidly erode its support for the war"; "He lamented what he said was the lack of strong evidence linking Iraq to the Sept. 11 terrorist attacks and proof of an imminent threat to American security"
3	Health	Vioxx drug	01/10/2004 - 07/10/2004	"Merck & Co. Inc., in a sudden admission of problems with a blockbuster drug, said yesterday that it was withdrawing its Vioxx arthritis pain medicine because of an increased risk of heart attack and stroke"; "The arthritis drug Vioxx might not be the only drug of its type that raises the risk of heart attack and stroke, scientists suggest in a report released yesterday by the New England Journal of Medicine"; "Problems with two widely used drugs — Mercks arthritis drug Vioxx and GlaxoSmithKlines Paxil, an antidepressant — raise red flags about the drug approval system"
4	Environment	Hurricane Katrina	02/09/2005 - 25/09/2005	"New Orleans today should be wounded but not mortally. Its people should be discomforted and distraught, but not dead or still at dire risk. Scientists at Louisiana State University say their computer models now suggest as many as 80,000 people may have perished"; "And then theres the fact that the leadership is pushing terrorism, and all the money goes to terrorism. We are certainly at risk of terrorism, but we are also at risk of earthquakes and winter storms and flooding, and there needs to be a balance"; "When New Orleans emerges from Katrinas floodwaters and begins to plan for its future, how will engineers and planners make the new city less susceptible to wind and water? "The really difficult question is, How much do you want to spend to make it how safe?" said John E. Durrant"
5	Business	Financial crisis	16/09/2008 - 07/10/2008	"Major financial institutions have teetered on the edge of collapse, and some have failed"; "With bad news gushing from the nations financial markets like water rushing over Missouris river banks, more Americans are confronting the prospect of living on less"; "The economic crisis and bailout are devastating to ordinary Americans"
6	Environment	Fukushima	13/03/2011 - 23/03/2011	"Two Japanese nuclear reactors whose cooling systems were knocked out following an earthquake and tsunami were joined Monday morning by a third reactor that lost its cooling system"; "California legislators at the state and federal levels are ratcheting up pressure on the Golden States two operating nuclear power plants - both, like Japans stricken Fukushima I, located in seismically active regions near the Pacific coastline"; "Anyone within striking distance of a nuclear power plant and an earthquake zone is asking the same question: Could it happen here?"
7	Geopolitics	US-Asia relations	10/08/2017 - 11/08/2017	"North Koreas threat on Thursday to test-fire ballistic missiles soon near the American territory of Guam deepened the challenge confronting the Trump administration"; "When President Trump used words of violence - "fire and fury the world has never seen" - to threaten North Korea, many people rushed to calm fears that his nuclear rhetoric might become a nuclear reality"; "Given the delicacy of the emerging security crisis, given the very real possibility of blundering into a catastrophic war, the President must learn to calm and focus his language"
8	Environment	Hurricane season	31/08/2017 - 08/09/2017	"As Southeast Texas residents emerged by the thousands from shelters, motels and friends' houses to return to flood-damaged homes, others remained in the grip of the crisis on Friday"; "With record-setting strength and speed, deadly Hurricane Irma is now within days of striking the southeastern U.S"; "What will happen when all that property is deluged by a megastorm, whether it is Irma or the hurricane after it? In six of Florida's most vulnerable cities, more than a million homes at risk for storm surge damage are not covered by flood insurance";

9	Health	COVID-19: Outbreak	11/02/2020 - 24/06/2020	"Countries across the world may now be faced with the task of limiting the spread of the disease on their own soil, not simply seeking to identify and quarantine infected people who had been in China"; "Should you wear a mask? Does airplane travel pose a greater risk? Do hand sanitizers work?"; "Italy on Monday became the first European country to announce severe nationwide limits on travel as the government struggled to stem the spread of a coronavirus outbreak that has hobbled the economy, threatened to overwhelm public health care and killed more people than anywhere outside China"; "Thousands of elderly residents of Minnesota nursing homes are being confined to their rooms, cut off from their relatives and gripped by fear of an invisible virus. Those who fall prey to the illness risk dying alone"
10	Entertainment	COVID-19: Impact	12/03/2020 - 16/08/2020	"The safety challenges created by COVID-19 place our football student-athletes at an unacceptable level of risk"; "Spectators must adhere to Health Department rules for indoor gatherings that call for social distancing, masking, and attendance limits"; "A dramedy about couples who are navigating the pandemic, it joins HBOs Coastal Elites, NBCs Connecting, and Netflixs Social Distance, all of which are set amid the Covid-19 crisis and were shot with all the strictures, safety precautions, and workarounds that such a crisis demands"
11	Domestic affairs	COVID-19: Impact	15/04/2020 - 17/04/2020	"What if you see her and you infect her with the illness, which then kills her? Won't that guilt be absolutely horrific?"; "On a recent workday, almost every business on Graham Ave. in Williamsburg, Brooklyn, was shuttered - keeping with the city's rules to stop the spread of coronavirus"; "President Trump made another head-turning claim of executive power in the middle of a briefing about the Covid-19 pandemic"
12	Health	COVID-19: Prevention	02/09/2020 - 17/12/2020	"The Centers for Disease Control and Prevention has notified public health officials in all 50 states and five large cities to prepare to distribute a coronavirus vaccine to health care workers and other high-risk groups as soon as late October or early November"; "Protections like wearing masks, which have proved divisive in the United States, are now widespread on the continent, helping people get on with their lives with calculated risk"; "Will we be able to trust the FDA when it approves the COVID-19 vaccine? Can we believe the CDC reports on COVID?"
13	Geopolitics	US withdraw from Afghanistan	14/04/2021 - 15/04/2021	"The withdrawal is calendar-based rather than conditions-based, which means the U.S. is withdrawing from Afghanistan not because conditions on the ground are conducive to peace and stability but because the war is unpopular domestically – and because Biden believes that the conflict with the Taliban is ultimately unwinnable"; "Drones, long-range bombers and spy networks will be used in an effort to prevent Afghanistan from re-emerging as a terrorist base to threaten the United States"
14	Health	COVID-19: Vaccine	14/04/2021 - 11/05/2021	"More than half of the population having had at least one shot means they have been extraordinarily successful"; "U.S. and European public health experts have emphasized that for most people, the benefits of the Covid vaccines far outweigh the risks. Several countries in Europe last week restricted use of the AstraZeneca-Oxford vaccine to older adults because rare cases of a blood disorder were occurring in younger people"; "Though it is unclear whether the vaccine was responsible for the clots, injections came to a sudden halt across the country"; "About 3 in 10 Republicans say they will definitely not get vaccinated, according to a Kaiser Family Foundation poll"

14 cases are identified as risk amplification signal events *within* the seven categories of risk news coverage. Key dates are the date range for which there is significantly high news coverage relating to the event. Sample news article text is a selection of text quotes from media reporting related to the risk event.

Table S3: US media risk topics (2000-2021)

	1: Human interest		2: R&D		3: Air safety		4: Financial markets		6: War		6: Pollution	
1	world	0.018	university	0.030	security	0.034	economy	0.032	war	0.019	plant	0.022
2	life	0.016	research	0.020	attack	0.015	market	0.024	Iraq	0.016	site	0.018
3	fact	0.015	study	0.018	airport	0.015	investor	0.015	attack	0.015	level	0.017
4	America	0.014	professor	0.016	airline	0.013	stock	0.015	Israel	0.013	water	0.013
5	today	0.011	center	0.014	plane	0.012	uncertainty	0.015	force	0.011	chemical	0.012
6	freedom	0.011	scientist	0.014	flight	0.012	price	0.014	troop	0.010	exposure	0.009
7	word	0.011	expert	0.014	passenger	0.011	growth	0.012	security	0.009	material	0.009
8	society	0.010	science	0.011	travel	0.010	rate	0.012	soldier	0.009	facility	0.009
9	history	0.009	question	0.011	homeland_sec	0.009	bond	0.012	Afghanistan	0.008	air	0.008
10	editor	0.009	director	0.011	pilot	0.009	investment	0.011	army	0.007	project	0.008
%		2%		1%		3%		4%		6%		3%

	7: Conflict		8: Environment disasters		9. Female health		10: Arts		11: Food safety		12: Financial management	
1	Iran	0.020	damage	0.024	woman	0.046	story	0.017	food	0.025	program	0.049
2	Russia	0.016	area	0.023	patient	0.039	show	0.013	product	0.017	money	0.032
3	war	0.016	water	0.022	hospital	0.038	book	0.013	store	0.016	plan	0.028
4	world	0.013	home	0.020	doctor	0.028	life	0.009	restaurant	0.011	cost	0.017
5	weapon	0.011	storm	0.013	surgery	0.012	love	0.008	consumer	0.010	budget	0.012
6	Washington	0.010	clim_change	0.012	procedure	0.012	film	0.008	customer	0.008	dollar	0.012
7	region	0.010	Florida	0.011	treatment	0.011	man	0.007	place	0.007	fund	0.011
8	Syria	0.009	region	0.009	care	0.011	art	0.007	item	0.006	project	0.011
9	agreement	0.007	river	0.008	health	0.010	movie	0.007	hand	0.006	benefit	0.010
10	talk	0.007	disaster	0.008	physician	0.009	character	0.006	bar	0.006	million	0.009
%		3%		3%		3%		5%		2%		2%

	13: Illness		14: Banking		15: Crime		16: Sports		17: Medicine		18: Job security	
1	study	0.024	bank	0.042	court	0.028	team	0.035	drug	0.056	worker	0.038
2	cancer	0.016	business	0.019	law	0.025	game	0.030	patient	0.019	employee	0.037
3	woman	0.014	firm	0.013	lawyer	0.017	player	0.027	product	0.017	job	0.036
4	disease	0.014	loan	0.013	prison	0.015	season	0.020	FDA	0.015	business	0.031
5	age	0.011	deal	0.013	lawsuit	0.014	sport	0.011	food_drug_ad	0.014	policy	0.022
6	man	0.010	money	0.011	charge	0.013	injury	0.010	market	0.012	cost	0.015
7	brain	0.009	investor	0.009	judge	0.013	league	0.009	medicine	0.011	insurance	0.014
8	body	0.008	sale	0.008	trial	0.011	field	0.009	medication	0.010	union	0.012
9	heart_disease	0.007	credit	0.008	attorney	0.010	fan	0.008	pharmacy	0.010	employer	0.011
10	researcher	0.007	investment	0.008	jail	0.010	play	0.008	doctor	0.009	coverage	0.011
%		4%		4%		4%		6%		3%		2%

Table presents the topics identified by the LDA topic modeling of risk-related news articles. See Section 3.2 for implementation details. The initial topic model reported 35 topics, but 3 topics were removed as non-risk topics, The table, therefore, reports the final 32 topics in the order in which the topic model reported them. The table reports the top 10 terms per topic, and their beta values - the statistical contribution of the term to the topic. The last row is the percentage of articles in the full dataset of 271,854 articles where the primary topic of the article belongs to that topic. Note: to allow the table to fit on the page the following terms were shortened: Topic 3, Term 9 'homeland_security' to 'homeland_sec'; Topic 8, Term 6 'climate_change' to 'clim_change'; Topic 17, Term 5 'food_drug_administration' to 'food_drug_ad'.

Table S4: US media risk topics (2000-2021) (contd.)

	19: Locality		20: Compliance		21: Child welfare		22: Education		23: European affairs		24: Road safety	
1	city	0.085	report	0.038	child	0.101	school	0.069	Europe	0.022	car	0.033
2	building	0.024	department	0.030	family	0.062	student	0.046	Britain	0.013	driver	0.023
3	home	0.022	agency	0.029	parent	0.037	district	0.021	border	0.013	road	0.022
4	fire	0.022	investigation	0.020	kid	0.022	university	0.019	europ_union	0.011	vehicle	0.020
5	resident	0.021	office	0.017	home	0.022	program	0.017	France	0.010	accident	0.012
6	county	0.020	letter	0.014	mother	0.016	education	0.015	Mexico	0.010	track	0.009
7	house	0.018	issue	0.012	life	0.014	Georgia	0.014	London	0.009	crash	0.009
8	neighborhood	0.015	problem	0.012	adult	0.014	teacher	0.014	Germany	0.008	injury	0.008
9	community	0.012	information	0.012	age	0.013	college	0.013	group	0.008	number	0.008
10	street	0.012	review	0.012	son	0.013	class	0.012	crisis	0.008	model	0.008
%		4%		1%		3%		3%		3%		3%

	25: Party politics		26: Policing		27: Cyber security		28: Tech failure		29: Regulation		30: Trade	
1	bill	0.029	police	0.034	information	0.022	foot	0.011	industry	0.027	China	0.043
2	election	0.021	officer	0.028	system	0.022	damage	0.011	rule	0.025	world	0.034
3	vote	0.020	man	0.027	technology	0.018	space	0.011	regulation	0.016	Japan	0.010
4	party	0.017	gun	0.015	datum	0.015	eye	0.010	oil	0.013	India	0.009
5	republican	0.015	violence	0.015	internet	0.013	ship	0.009	standard	0.011	Hong_Kong	0.008
6	democrat	0.014	victim	0.014	network	0.012	hour	0.008	change	0.011	effort	0.008
7	issue	0.013	death	0.012	security	0.012	line	0.008	requirement	0.009	trade	0.008
8	lawmaker	0.013	crime	0.011	service	0.012	crew	0.008	process	0.008	Beijing	0.008
9	voter	0.012	church	0.011	computer	0.011	minute	0.007	law	0.008	deal	0.007
10	campaign	0.012	person	0.010	access	0.010	mission	0.007	Pennsylvania	0.007	nation	0.007
%		4%		3%		3%		2%		2%		2%

	31: Elections		32: Infectious disease	
1	president	0.061	virus	0.034
2	administration	0.025	vaccine	0.029
3	white_house	0.023	coronavirus	0.016
4	trump	0.020	health	0.014
5	Washington	0.020	disease_control	0.011
6	congress	0.017	disease	0.010
7	mr_trump	0.016	prevention	0.010
8	national_sec	0.014	infection	0.010
9	nation	0.013	center	0.009
10	American	0.013	mask	0.008
%		3%		4%

Table presents the topics identified by the LDA topic modeling of risk-related news articles. See Section 3.2 for implementation details. The initial topic model reported 35 topics, but 3 topics were removed as non-risk topics, The table, therefore, reports the final 32 topics in the order in which the topic model reported them. The table reports the top 10 terms per topic, and their beta values - the statistical contribution of the term to the topic. The last row is the percentage of articles in the full dataset of 271,854 articles where the primary topic of the article belongs to that topic. Note: to allow the table to fit in the page the following terms were shortened: Topic 23, Term 4 'european_union' to 'europ_union'; Topic 31, Term 8 'national_security' to 'national_sec'.

Figure S1: Timeline of business risk news. The top figure shows the per-topic timeline, bottom figure shows trends and signal events in the timeline. See Section 3.3 for further details on the construction of the bottom figure.

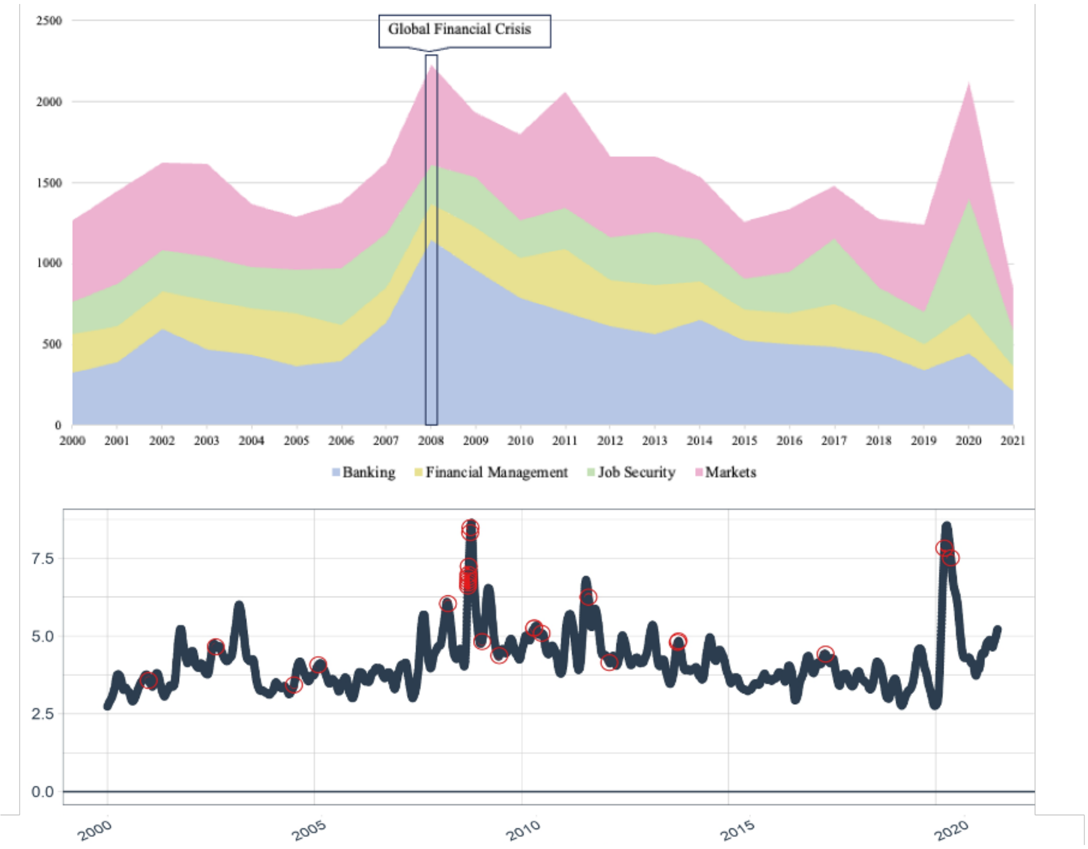

Figure S2: Timeline of domestic affairs risk news. The top figure shows the per-topic timeline, bottom figure shows trends and signal events in the timeline. See Section 3.3 for further details on the construction of the bottom figure.

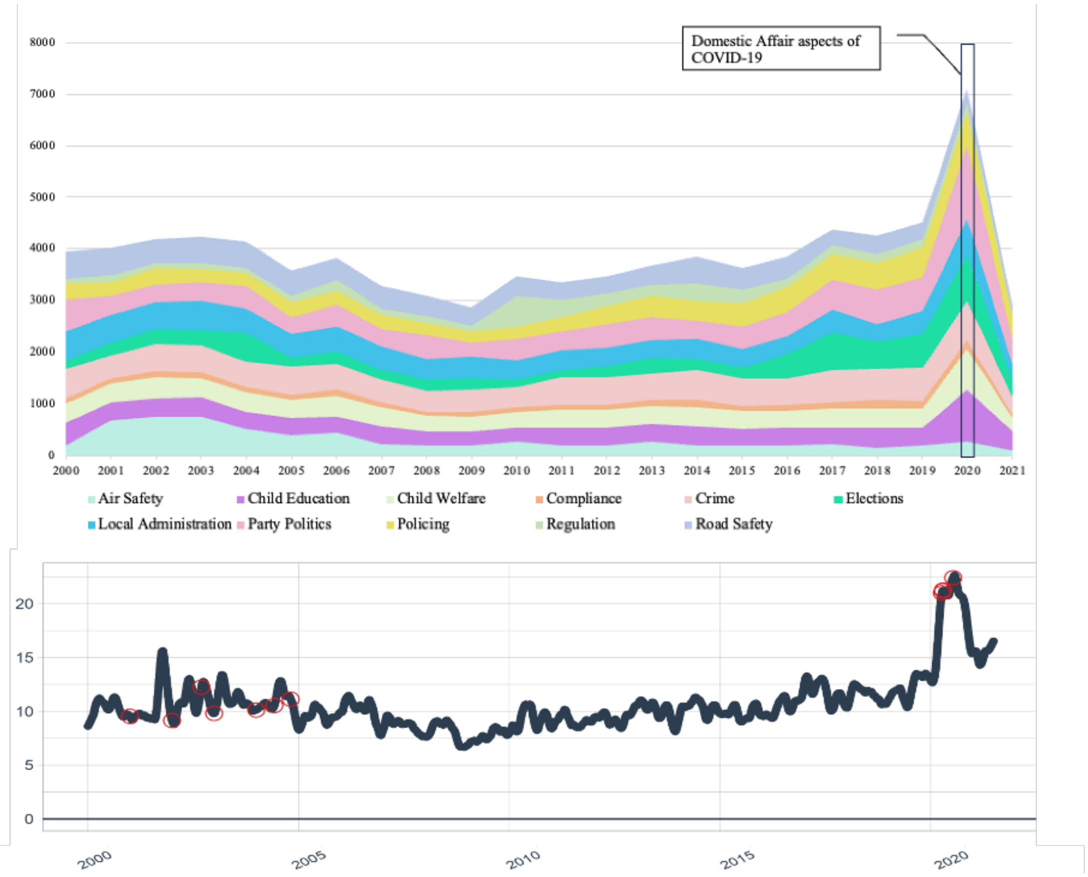

Figure S3: Timeline of entertainment risk news. The top figure shows the per-topic timeline, bottom figure shows trends and signal events in the timeline. See Section 3.3 for further details on the construction of the bottom figure.

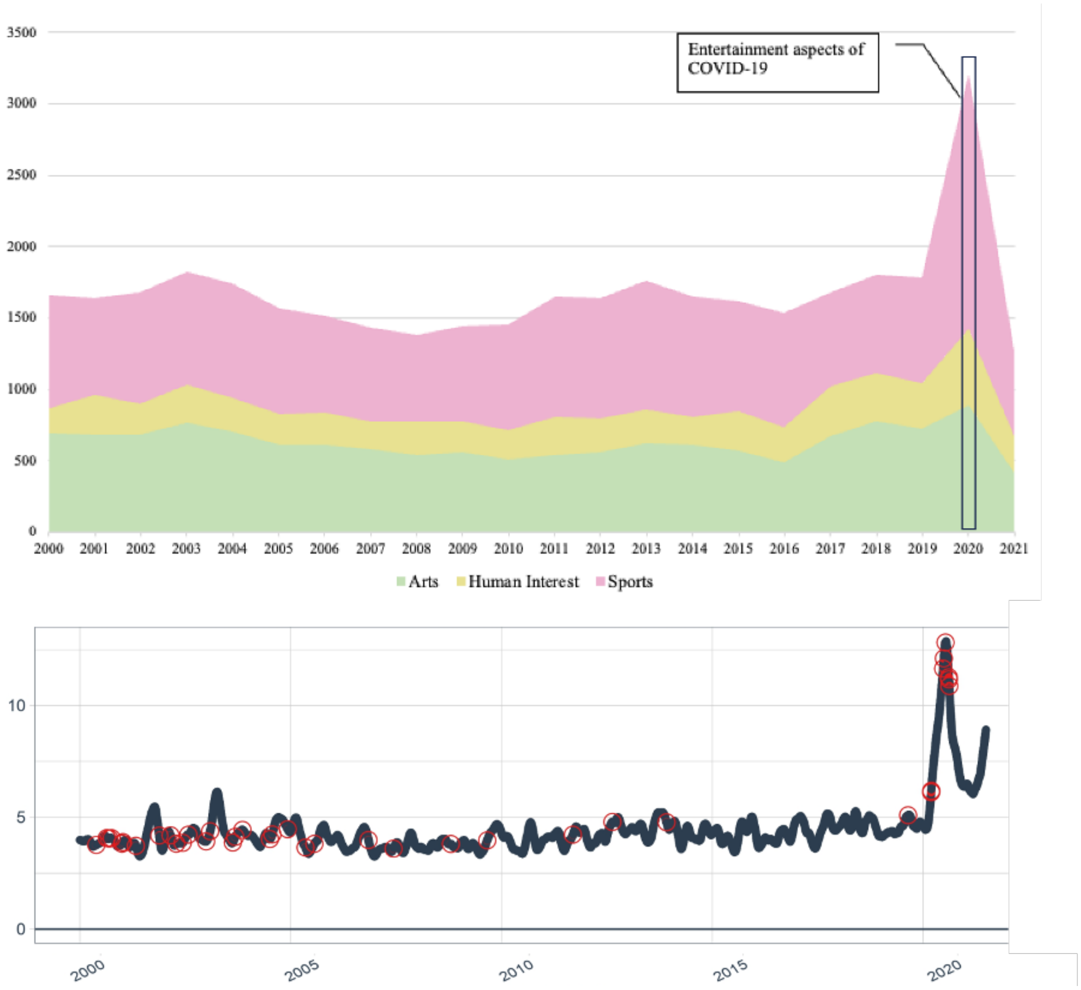

Figure S4: Timeline of environmental risk news. The top figure shows the per-topic timeline, bottom figure shows trends and signal events in the timeline. See Section 3.3 for further details on the construction of the bottom figure.

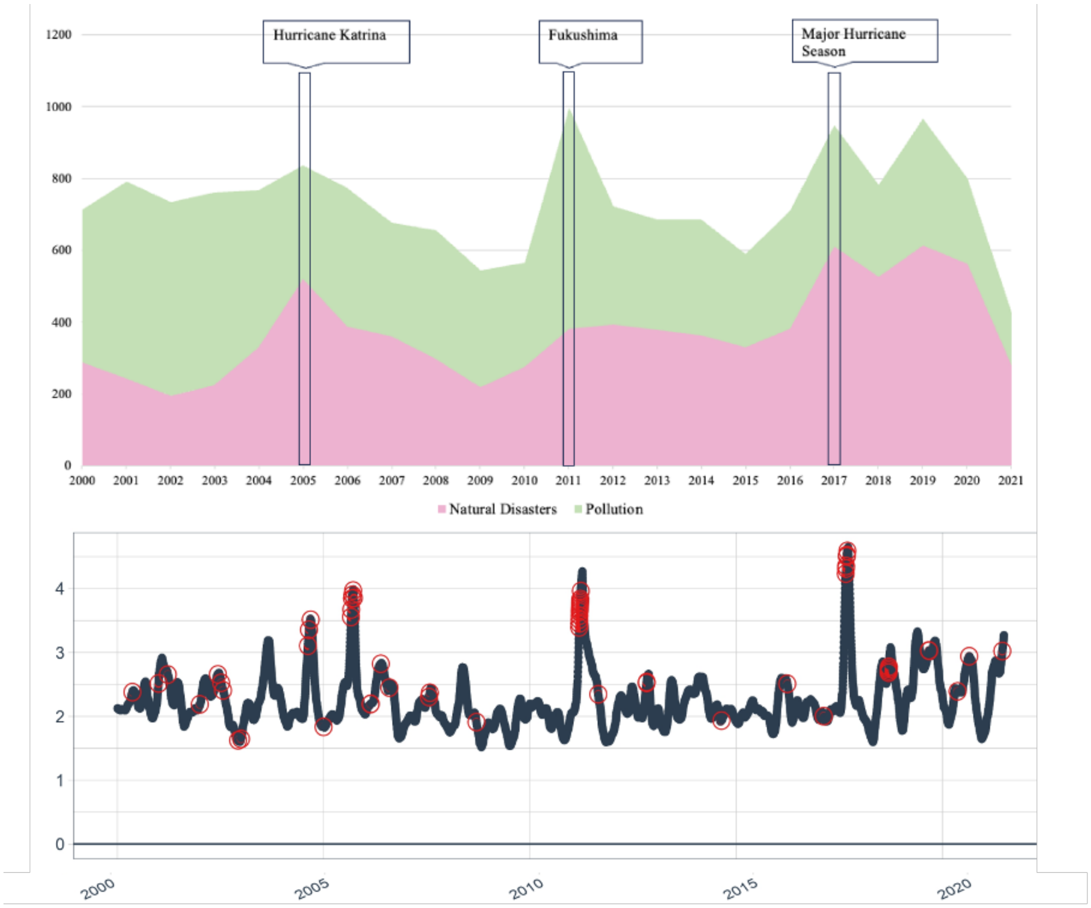

Figure S5: Timeline of technology risk news. The top figure shows the per-topic timeline, bottom figure shows trends and signal events in the timeline. See Section 3.3 for further details on the construction of the bottom figure.

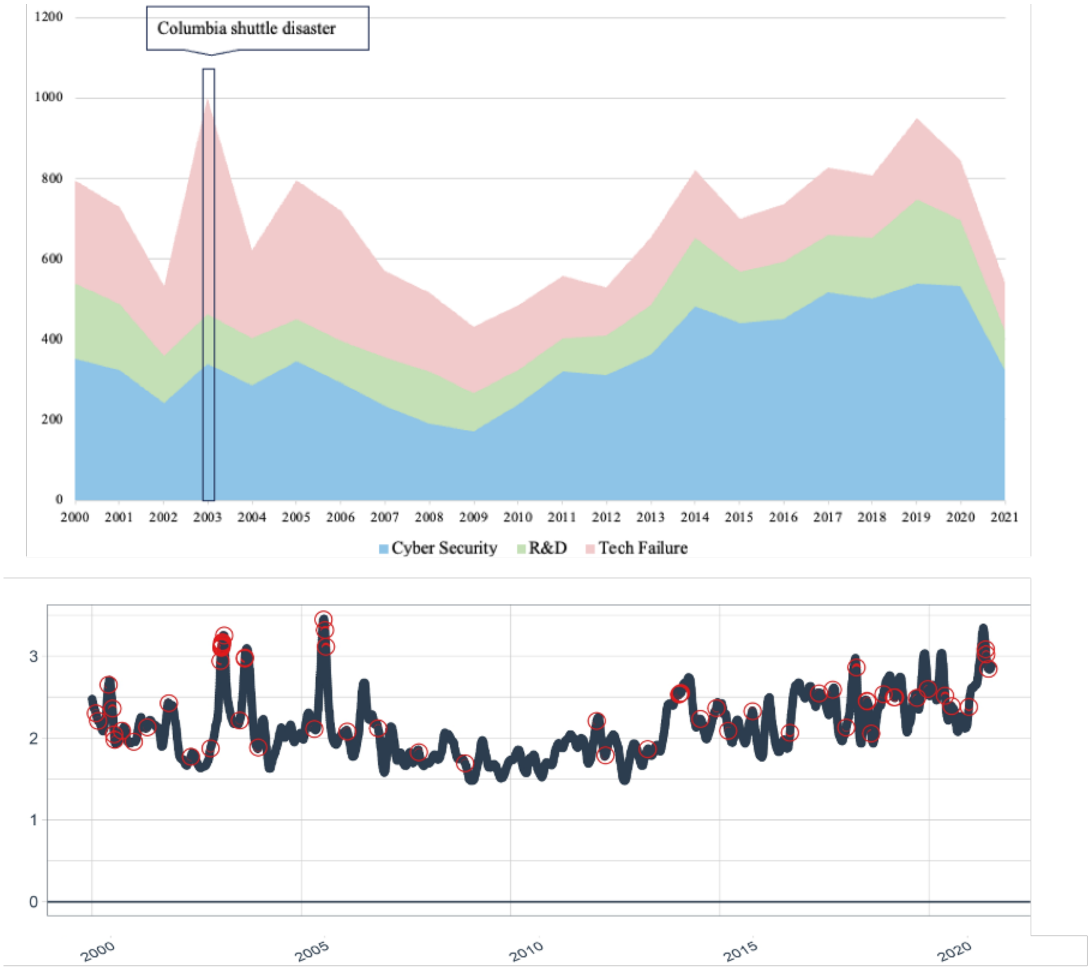

Figure S6: Timeline of geopolitical risk news. The top figure shows the per-topic timeline, bottom figure shows trends and signal events in the timeline. See Section 3.3 for further details on the construction of the bottom figure.

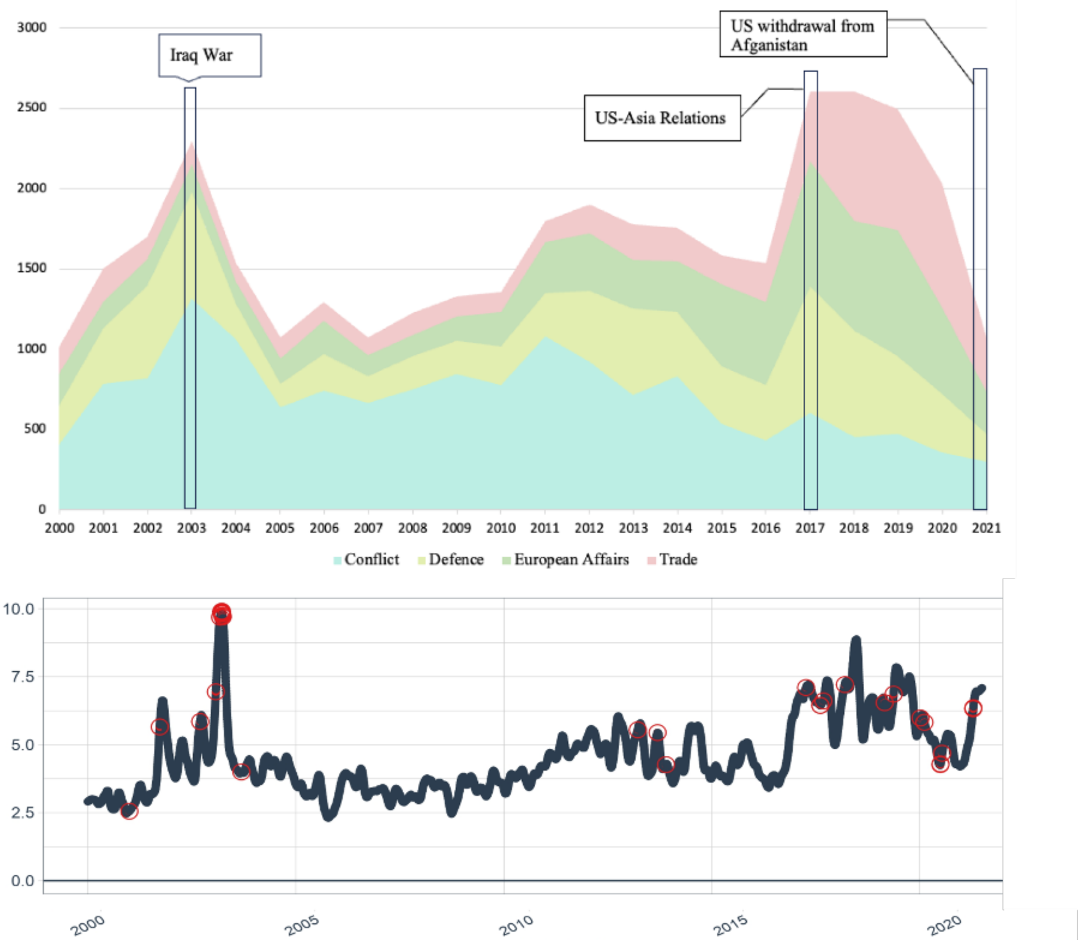

Figure S7: Timeline of health risk news. The top figure shows the per-topic timeline, bottom figure shows trends and signal events in the timeline. See Section 3.3 for further details on the construction of the bottom figure.

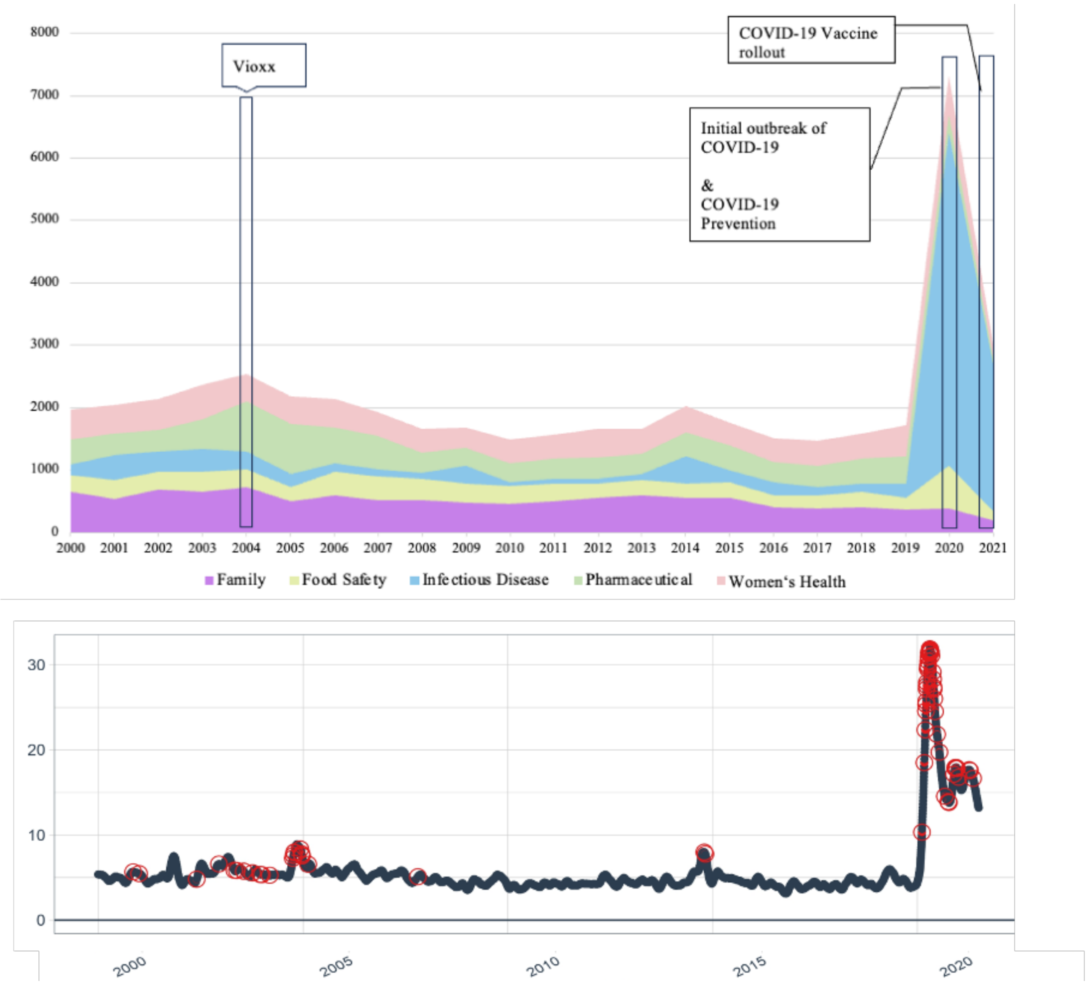

Supplement: Supplementary file 1 — Table S1: Risk‐related news dataset description Table S2: Major risk amplification events featured in US news media Table S3: US media risk topics (2000‐2021) Table S4: US media risk topics (2000‐2021) (contd.) Figure S1: Timeline of business risk news. Figure S2: Timeline of domestic affairs risk news. Figure S3: Timeline of entertainment risk news. Figure S4: Timeline of environmental risk news. Figure S5: Timeline of technology risk news. Figure S6: Timeline of geopolitical risk news. Figure S7: Timeline of health risk news. [file RISA-45-1940-s001.pdf]
